# Valence Asymmetry in Cognition—A Formal Account

**DOI:** 10.1177/10888683251407820

**Published:** 2026-03-18

**Authors:** Nele Freyer, Christian Unkelbach, Anne Wiedenroth, Hans Alves, Paula Knischewski, Daniel Leising

**Affiliations:** 1 Technische Universität Dresden, Germany; 2 Universität zu Köln, Germany; 3 Ruhr-Universität Bochum, Germany

**Keywords:** valence asymmetry, formalization, differential processing, negativity bias

## Abstract

**Academic Abstract:**

*Valence asymmetries*—the tendency for bad stimuli to elicit more processing effort than good ones—have been widely observed but remain theoretically contested. To advance this debate, we present a formalized account integrating two major explanatory perspectives: the *intrapsychic* (or *phylogenetic*) approach, which locates the effect in internal evaluative mechanisms, and the *ecological* (or *ontogenetic*) approach, which attributes it primarily to environmental factors. We introduce a concise set of parameters to specify key concepts and analyze the argumentative structure of each perspective. This yields three major insights: (a) the traditional labels for these approaches are misleading, and we suggest using *valence-driven* and *distinctiveness-driven* instead, (b) theories must specify how exactly *good* and *bad* stimuli are defined, and (c) some explanations rely on implicit yet critical assumptions, such as the probability of having contact with stimuli. Clarifying these foundations provides a framework for informative empirical tests in future research.

**Public Abstract:**

Why do people pay more attention to bad things than to good ones? Psychologists call this pattern a *valence asymmetry*. Although the effect is well established, its causes are still debated. To advance this debate, we translate two leading ideas about this bias into a precise mathematical model. This allows us to see how the explanations differ, what each predicts, and where they overlap. This analysis reveals three important insights: First, some widely used terms are misleading, and we suggest using clearer alternatives like *valence-driven* and *distinctiveness-driven* instead. Second, researchers need to define more carefully what actually counts as *good* or *bad*. Third, many theories rely on hidden assumptions—such as how often people encounter certain kinds of stimuli. Making those assumptions explicit should help future studies test competing explanations more directly.

## Introduction

Negative and positive stimuli differ in the amount of processing effort they elicit. These *valence asymmetries* in cognition in general and social cognition in particular have been a longstanding topic of discussion in psychological research. Processing differences between positive and negative information have been shown in numerous studies: For example, negative stimuli draw more attention (Pratto & John, 1991), are detected more readily (e.g., Nasrallah et al., 2009), and carry greater weight in the aggregation of information (Anderson, 1965). Together, these and other findings led Baumeister et al. (2001) to conclude that “bad is stronger than good” (p. 323). Such negativity biases have important implications across applied domains, from economic decision making (e.g., “losses loom larger”), media (e.g., “if it bleeds, it leads”), to clinical psychology (e.g., post-traumatic stress disorder). At the same time, some research has also identified instances of positivity biases, such as a faster processing of positive stimuli (Unkelbach et al., 2008; 2010; Unkelbach, 2012).

Different explanations for such valence asymmetries point to phylogenetic roots (Baumeister et al., 2001; Rozin & Royzman, 2001), motivational factors (Taylor, 1991), separate processing systems (Cacioppo & Berntson, 1994; Peeters, 1971), or ecological influences (Unkelbach et al., 2020). However, integrating, comparing, and critically testing these explanations is a challenge, as the example of the phylogenetic and ecological explanations reveals.

According to the *phylogenetic approach*, also known as *evolutionary* or *intrapsychic*, valence asymmetries in processing effort are rooted in evolution: For various reasons, it may have been adaptive to spend more processing effort on negative compared to positive stimuli in past generations (Baumeister et al., 2001; Rozin & Royzman, 2001). Thus, by means of natural selection, humans developed a tendency to attend more to negative stimuli, remember them better, and more easily retrieve them when necessary.

An alternative *ecological*, or *ontogenetic*, *approach* is that bad stimuli are not processed differentially because of their valence but because they happen to be more distinct than good ones in people’s standard environment (Unkelbach, Koch, & Alves, 2019; Unkelbach, Alves, & Koch, 2020). Hence, differential processing is not explained by valence-dependent intrapsychic but by ecological mechanisms (see Fiedler, 2014).

In an ideal scientific world, one would now go about testing these two approaches against each other. However, at the present stage, this endeavor is difficult, if not impossible, because of the narrative nature of the explanations. Although narrative theories that express their key ideas in natural language terms predominate and are even incentivized in psychology (Frankenhuis et al., 2023), they come with serious limitations (Glöckner & Betsch, 2011; Leising et al., 2022; Scheel, 2022; Sell, 2018). Their relative lack of clarity and precision makes it hard to delineate the critical predictions and potentially disprove them. Further, it often remains obscured how different theories relate to one another. Sometimes, two theories or concepts described as distinct may in fact be structurally identical, but this goes unnoticed because they use different terminology to describe that structure.

To enable informative tests, theories must be both specified and integrated into overarching frameworks (Glöckner & Betsch, 2011; Muthukrishna & Henrich, 2019). The best way to do that is by developing a formal model. Formal modeling involves re-formulating narrative ideas in clearer, formal-logical terms (for examples of different techniques, see Borsboom et al., 2021; Glöckner & Betsch, 2011; Smaldino et al., 2015). Expressing concepts in this common language allows delineating similarities and differences between approaches and identifying the elements that distinguish one explanation from the other. In addition, formalization forces researchers to make implicit assumptions explicit. Ultimately, formal modeling allows informative empirical designs and precise predictions that are capable of falsifying entire explanations or specific components thereof (Borsboom et al., 2021; Scheel, 2022).

The present aim is to develop a formal model that describes the phylogenetic (intrapsychic) and the ecological (ontogenetic) approaches to valence asymmetries. By specifying these accounts in terms of a common set of variables, we clarify their key similarities and differences and lay the groundwork for empirical tests capable of distinguishing between them. In our reporting, we draw on the checklist of indicators for theoretical rigor proposed by Lange et al. (2025) as a guiding framework. Before we start introducing the model’s parameters, we first clarify a few foundational ideas.

### Defining the Phenomenon

As a first step, the phenomenon needs to be defined and separated from possible explanatory mechanisms. The term *valence asymmetry* is an umbrella term for a wide range of cognitive phenomena following the central pattern that “bad is stronger than good” (Baumeister et al., 2001, p. 323). People detect negative stimuli more readily and discriminate them more accurately (Hansen & Hansen, 1988; Nasrallah et al., 2009; Pratto & John, 1991), process them more deeply (Ito et al., 1998), and remember them better in recognition (Ortony et al., 1983; Robinson-Riegler & Winton, 1996) and free recall tasks (Brown & Kulik, 1977). Negative traits and behaviors also carry more weight in impression formation (Anderson, 1965; Feldman, 1966; Fiske, 1980). In decision making, negative outcomes are weighed more heavily (Kahneman & Tversky, 1979), and actions with negative consequences are seen as more intentional.

However, there are also positivity advantages in some domains: Positive stimuli are processed faster in classification (Bargh et al., 1992) and lexical decision tasks (Unkelbach, Von Hippel, et al., 2010), are associated more easily (Anisfeld & Lambert, 1966), and are rather seen as congruent with other stimuli (Anselmi et al., 2011; Bar-Anan et al., 2009).

Despite this variety, most theoretical approaches do not distinguish between single phenomena but aim to find one common explanation for them all. Even though others emphasize that different phenomena might require different explanations (see Rozin & Royzman, 2001), we aim for a model that explains valence asymmetries as a whole. Given the range of relevant phenomena, this will necessarily require some simplifications. From our perspective, the most concise way to summarize most of the stimulus-related asymmetries in the processing of good and bad stimuli is to frame them in terms of higher *processing effort* for bad stimuli than for good ones: This encompasses not only the negativity biases of greater attention, deeper processing, and better recall for bad stimuli, but also the positivity bias of faster processing for good stimuli.

### Separating the Approaches

As mentioned, we will focus on two strands of theorizing about valence asymmetry that have been referred to as intrapsychic, phylogenetic, or evolutionary for the earlier approach (e.g., Baumeister et al., 2001; Rozin & Royzman, 2001), versus ecological or ontogenetic for the more recently developed approach (e.g., Alves et al., 2017; Unkelbach, Koch, & Alves, 2019; Unkelbach, Alves, & Koch, 2020).

By contrasting *intrapsychic* with *ecological* approaches, previous accounts highlighted the difference that valence asymmetry phenomena may be rooted more strongly in the typical perceiver’s disposition to expend more effort on the processing of certain types of stimuli as compared to other types (intrapsychic), or in the actual distribution of stimuli in the environment (ecological). Upon closer inspection, however, this distinction becomes more apparent than real because both theoretical frameworks require assuming a disposition on how to process certain types of stimuli preferentially, as well as making assumptions about the environment, as we will show later.

Other naming conventions, such as *phylogenetic* (or *evolutionary*) versus *ontogenetic*, highlighted the idea that valence asymmetry phenomena may be rooted more strongly in the developmental history of our species (phylogenetic), or in the learning history of the individual (ontogenetic). Again, this distinction is misleading because both approaches contain assumptions about evolution: The environment that results in differential processing according to the ontogenetic approach is also assumed to be shaped by evolution (Unkelbach, Alves, & Koch, 2020, referring to Axelrod & Hamilton, 1981; Denrell, 2007).

We will therefore replace the naming tradition highlighted above with one that more precisely captures the key difference between the two approaches: We will call the first (intrapsychic, phylogenetic) approach *valence-driven*(referring to Baumeister et al., 2001; Rozin & Royzman, 2001) and the second (ecological, ontogenetic) one *distinctiveness-driven* (referring to, e.g., Alves et al., 2017; Unkelbach, Alves, & Koch, 2020). Within the valence-driven approach, people expend greater effort on the processing of negative stimuli because stimulus negativity signals greater relevance. Within the distinctiveness-driven approach, people expend greater effort towards stimuli that are distinct relative to average stimuli (e.g., present or in memory). In a typical environment, distinctive stimuli happen to be mostly negative, but that negativity does not by itself explain valence asymmetry. In an environment in which stimulus valence and stimulus distinctiveness are uncorrelated, no valence asymmetries should occur.

### Explanatory Mechanisms

Within both approaches, several explanatory mechanisms are proposed, which currently lack precision and clarity in the respective literature. Clarifying these mechanisms is a major task and benefit of formal modeling.

#### Valence-Driven Approach

The valence-driven approach proposes several mechanisms to explain an adaptive advantage of differential processing: The main argument by Rozin and Royzman (2001) is the higher *potency* of bad stimuli as compared to good ones. Negative events are assumed to be “more threatening than are positive events beneficial” in terms of survival and procreation (Rozin & Royzman, 2001, p. 314). A further argument is the so-called *Chain Principle*, originally by Weinberg (1975), used as an explanation for valence asymmetries by Baumeister et al. (2001): A system is only as good as its weakest component. This need for consistency is not only assumed across different elements of a system but also across time. The required consistency across time is explained by the *asymmetry of life and death*: “The individual remains alive after several years only if he or she managed to survive every single day, and no degree of optimal experience on any given day can offset the effects of failing to survive on another” (Baumeister et al., 2001, p. 358).

However, due to their ambiguous phrasing, it is unclear how these mechanisms relate to each other and to the phenomenon itself. This makes it challenging to examine their interplay within the valence-driven approach and almost impossible to compare them with the distinctiveness-driven approach. Note that there are even more arguments such as the greater need for change signaled by bad stimuli (see Baumeister et al., 2001, p. 357), their higher informational complexity (Rozin & Royzman, 2001, p. 314), their higher contagion (Rozin & Royzman, 2001, p. 314), and a higher required rapidity of response to negative stimuli (Rozin & Royzman, 2001, p. 314). Nonetheless, we will focus on the mechanisms we consider most essential to the explanations.

#### Distinctiveness-Driven Approach

The distinctiveness-driven approach assumes that, in a typical environment, bad stimuli are more *distinct* than good ones in two respects: (a) their lower *frequency* (e.g., Boucher & Osgood, 1969; Matlin & Stang, 1978; Peeters, 1971, Unkelbach, Koch, & Alves, 2019) and (b) their lower *similarity* (e.g., Alves et al., 2017; Koch et al., 2016; Unkelbach, Fiedler, et al., 2008; see Unkelbach, Alves, & Koch, 2020, for a review). While good stimuli are more frequent and similar to each other, bad stimuli are rarer and more diverse.

This has been explained with the so-called *Range Principle*, that is, the idea that life is only possible within a narrow range of environmental states (e.g., Alves et al., 2017). Hence, there might be a *too little* and a *too much* of most stimulus characteristics. Rather moderate stimuli in the center of a scale are good, whereas more extreme stimuli in the outer ranges of the scale are bad. For instance, to ensure survival, temperatures must neither be too cold nor too hot. In combination with the assumption of normally distributed stimulus characteristics (Unkelbach, Fiedler, et al., 2008; Unkelbach, Alves, & Koch, 2020), good stimuli would then be more frequent as well as more similar than bad ones in a typical environment. Another argument is the so-called *Anna Karenina Principle* (Diamond, 1997): For a state or stimulus to be positive, all its dimensions must be jointly positive, whereas as soon as a stimulus is bad in one dimension, the whole stimulus is bad (Unkelbach, Alves, & Koch, 2020). We will later see that this mechanism is basically the same as what Baumeister et al. (2001) call the Chain Principle. Taken together, this is assumed to not only explain the negativity biases subsumed as valence asymmetries but also the emergence of positivity biases, such as the faster processing of positive stimuli (e.g., Unkelbach, Von Hippel, et al., 2010).

One challenge regarding these mechanisms is a conflation between concepts and operationalizations. While the concepts (e.g., similarity, frequency) denote assumptions about the external environment and are therefore objective, their operationalization in studies is often subjective (e.g., Alves, Unkelbach, et al., 2015; Alves, Koch, & Unkelbach, 2016; Koch et al., 2016), for instance, when relying on the spatial arrangement method (see Goldstone, 1994). In our formal conceptualization, we are interested in objective frequency and similarity that do not involve any subjective evaluation.

### The Problem with Good and Bad

Lastly, but very crucially, both approaches and virtually all other accounts on valence asymmetry lack a clear definition of *good* and *bad*. Some authors use no definition at all (e.g., Rozin & Royzman, 2001), others define only either good or bad (e.g., Taylor, 1991), and others define good and bad in other, not further specified terms such as “desirable, beneficial, or pleasant” and “undesirable, harmful, or unpleasant” (Baumeister et al., 2001, p. 324 f.). Unkelbach, Alves, and Koch (2020) define the valence of a stimulus by how much it is in line with an organism’s “goals and needs” (p. 117) following a Lewinian approach (Lewin, 1943). In all these approaches, it ultimately remains unclear what exactly separates good from bad stimuli. It is inherent in most accounts that stimuli are not only good or bad but can be so to a certain degree, that is, there is at least one valence dimension. However, it is not clarified where exactly the threshold lies between good and bad, and if that threshold is stable or might vary, depending on the frame of reference (as suggested by Parducci, 1965).

There is again some confusion concerning subjectivity or objectivity. Most empirical studies define valence as a subjective evaluation, while the theoretical reasoning refers to an objective entity. Baumeister et al. (2001) as well as Rozin and Royzman (2001) closely link the label bad with death. Unkelbach, Alves, and Koch (2020) also refer to survival to justify their range principle, stating that “human life is only possible in a very narrow range” (p. 157). In our formal account, we aim for a definition that may inform, but exists in principle, independent of subjective evaluations as delineated in the following.

## Formal Model

The following formal model contains the key variables from both narrative explanations, but also variables not explicitly included but necessary to enhance precision. By formalizing the narrative concepts, we can examine under which assumptions the proposed mechanisms reproduce the empirical phenomena. A main insight will be that we can model the valence-driven and distinctiveness-driven approaches with the same set of parameters and thus compare them with each other.

A table that lists all these parameters and an overview of the key aspects in the model are provided in the Appendix. Note that formalization requires keeping a balance between the complexity inherent in reality and the parsimony necessary for informative models (i.e., abstractions from reality). Thus, we introduced several simplifications, which are also listed in the Appendix. To support the model’s logical consistency and provide instructive figures, we conducted simulations in R (v 4.3.3, R Core Team, 2024). We describe these in the online supplement (osf.io/e3y65), which also provides access to commented scripts. For vizualization, we used ggplot2 (Wickham, 2016) and plotly (Sievert, 2020). ChatGPT was used to support creating the figures and to optimize the scripts.

We start by defining the environment in which a perceiver is placed and the stimuli that they encounter. We ignore any inter-individual differences between perceivers and only regard them as the average person. First, we assume that the perceiver is completely passive. Later, we will introduce the possibility of processing stimuli with a certain processing mechanism. Environments differ systematically in several aspects, which affects the adaptive advantage of such processing mechanisms. A crucial element in our model will be the consequence, or outcome, of encountering and processing stimuli. While in reality, there are many different possible consequences, the only relevant consequence in our model is the probability to survive P(L) (*L* for *live*). We chose this outcome because it plays such a crucial role in both explanations.

### The Environment

The environment is extremely simplified and only consists of stimuli that the perceiver encounters one after another. Stimuli can be everything from inanimate objects to animals or people to natural phenomena. We assume a finite set N of stimuli. By encounter, we mean that the perceiver is close enough to a stimulus to detect its properties, but not yet close enough to be affected by it. Imagine a perceiver who sits in a park and spots a dog.

We distinguish two cases for this encounter: The dog can either approach the perceiver or just walk along its way. More broadly speaking, encountered stimuli can either get in contact with the perceiver or pass by, indicated by C for *contact* and $\bar{C}$ for *no contact*. This distinction is crucial because it fundamentally affects the outcome. We want to model that not only getting in contact with a stimulus is consequential but missing it will be as well.

Both cases, contact and no contact, come along with a certain *survival probability*. The survival probability when getting in contact with the dog depends on its properties, for instance, how aggressive it is. We call these properties *substance levels*. Strictly speaking, we understand substance as a non-verbal property of a stimulus that exists independent of any subjective evaluation (following Leising et al., 2015), but we use the term *aggressive* here as an illustrative example.

To simplify things, we will treat stimuli as unidimensional for now. That is, we assume that all stimuli vary along the same substance dimension (i.e., aggressiveness), and this is the only dimension that matters to the perceiver. We denote a substance dimension as S with the substance levels of single stimuli being s . Assuming most substance dimensions are continuous and bounded to meaningful minima and maxima, we ignore possible nominal dimensions.

If the dog were extremely aggressive, it would kill the perceiver. If, in contrast, the dog were calm, it would not pose a danger. We denote this survival probability of having contact with a stimulus that is associated with its substance level as P(L|C,S=s) and call it *survivability*. The relationship between substance levels and the survival probability when getting in contact is the *survivability function* l(s) which is displayed in Figure 1. This function determines the probability of surviving getting in contact with a stimulus for every possible substance level. This function will be crucial when we later distinguish good from bad stimuli. Conceptually, the survivability function may have any shape. We will only regard linear (Figure 1A) and invertedly *U*-shaped (concave, Figure 1B) functions. In our example, the survivability function would be roughly linear, with a negative slope: The more aggressive a dog is, the lower the probability of surviving the contact with it.

**Figure 1.**
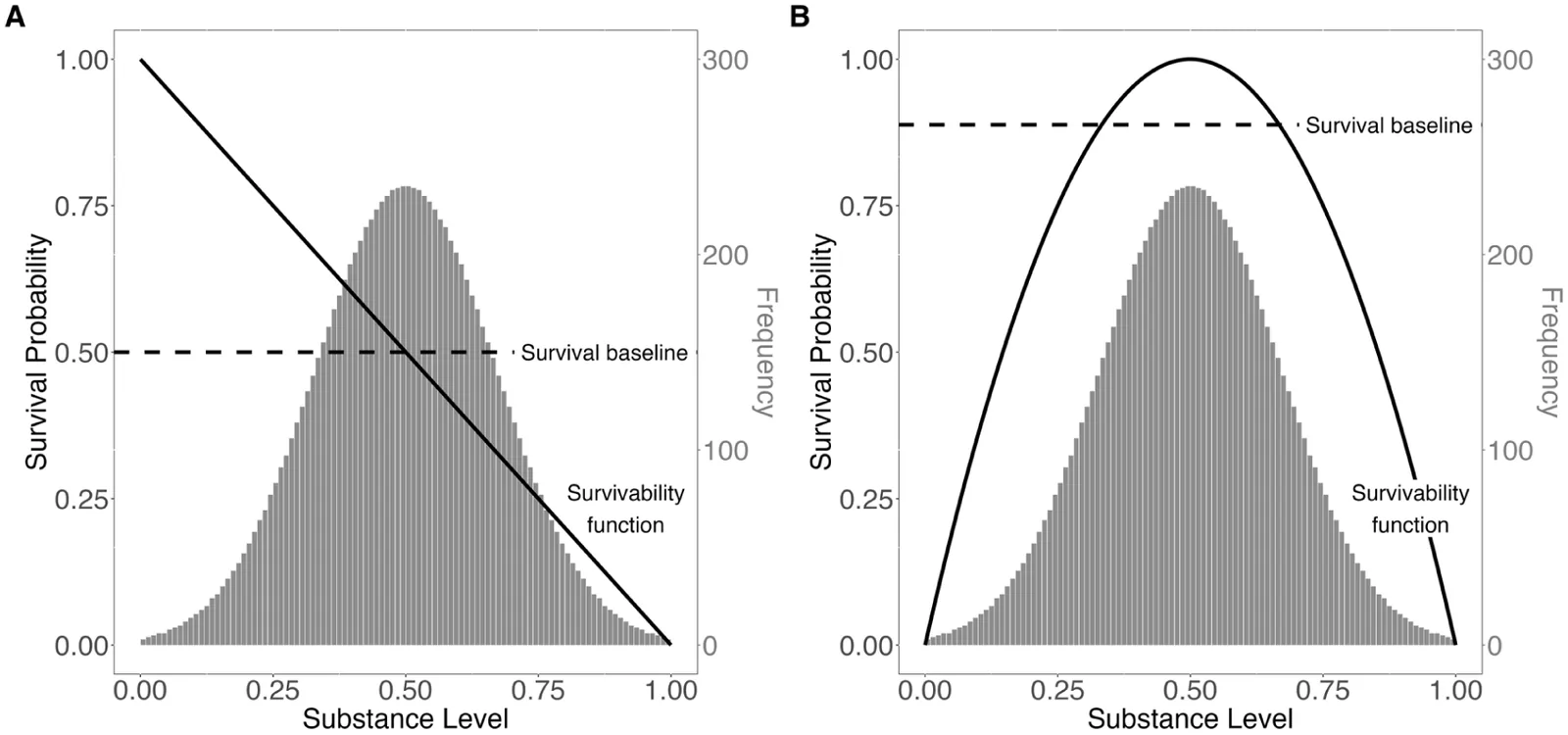
Survival probability. *Note.* Gray bars indicate the substance level distribution $f_{S}\left(s\right)$ . Panel A shows a negative linear survivability function l(s) and an absolute survival baseline of $P\left(L|\bar{C}\right)=0.5$ . Panel B depicts a concave survivability function l(s) and a relative survival baseline. Substance levels are normalized to range from 0 to 1.

The *substance distribution*, $f_{S}(s)$ , reflects how the substance levels of the stimuli are distributed within a given environment. In our example shown in Figure 1A, these levels follow a normal distribution. In contrast, in a kennel of fighting dogs, most encountered dogs would be aggressive, whereas in the park, most dogs would be rather peaceful—resulting in left- and right-skewed distributions, respectively. Because the survivability of a stimulus depends on its substance level, the distribution of substance levels determines the corresponding distribution of survivability values.

Not having contact with a stimulus does not automatically guarantee survival either. For instance, the environment might still be hostile, and life-sustaining stimuli might be missed. Think of a person in the desert, missing a life-saving spring. In our dog example, being accompanied by a peaceful dog might actually be beneficial in terms of survival, whereas not having contact with it exposes the perceiver to the dangers of the environment.

The survival probability when not having contact with a stimulus should be independent of its characteristics. We call this the *survival baseline* and denote it as $P(L|\bar{C})$ . It is represented by a dashed line in Figure 1. There are different ways in which a specific value for the survival baseline $P(L|\bar{C})$ may be specified: First, one may simply choose some *absolute* value, like .5 (as in Figure 1A). This reflects the idea that not getting in contact with any stimulus neither promotes survival nor death. Second, one may use a value that is *relative* to the environment, for example, the expected survivability P(L|C) , which is the aggregated survivability of the substance levels of all stimuli in the given environment (as in Figure 1B). This is the type of survival baseline that we implied in our examples above. In the desert, this baseline would be quite low, whereas in a park with mostly peaceful dogs, it would be pretty high.

In reality, both the survivability function and the survival baseline may not only vary from one environment to another but also across perceivers and over time (e.g., depending on the current state of the perceiver). Incorporating a state-specific survival baseline would be interesting but also come with a much higher level of complexity. We hence ignore it here, though we return to this point in our discussion.

### Valence

To examine explanations of valence asymmetries, we need to clearly distinguish *good* from *bad* stimuli. In other words, we need to identify the *valence* V(S=s) of a stimulus. In our model, we can make this distinction by comparing the probability of survival when having contact with the stimulus P(L|C,S=s) and the probability of survival when not having contact with the stimulus $P(L|\bar{C})$ . This leads to valence values that can range between 1 and −1, depending on the position of the survival baseline.

(1) $$V(S\;=\;s)=P(L|C,\;S=s)-P(L|\bar{C})$$

If this difference between the survivability of a stimulus and the survival baseline, that is, the valence of the stimulus, is positive, then the stimulus is good, which we denote as G , and if it is negative, the stimulus is bad, indicated by $\bar{G}$ , as depicted in Figure 2. The survival baseline functions like a threshold between good and bad stimuli. This conceptualization translates the idea of good stimuli being beneficial and bad stimuli being harmful (Baumeister et al., 2001) to formal terms: A stimulus is beneficial and therefore good when the outcome of getting in contact with it is better than not getting in contact. Vice versa, it is harmful and thus bad when the outcome of missing or avoiding it is superior to having contact with it. It also results in at least one range of good and one range of bad stimuli, which will be important when analyzing the range principle later on (Alves et al., 2017).

**Figure 2.**
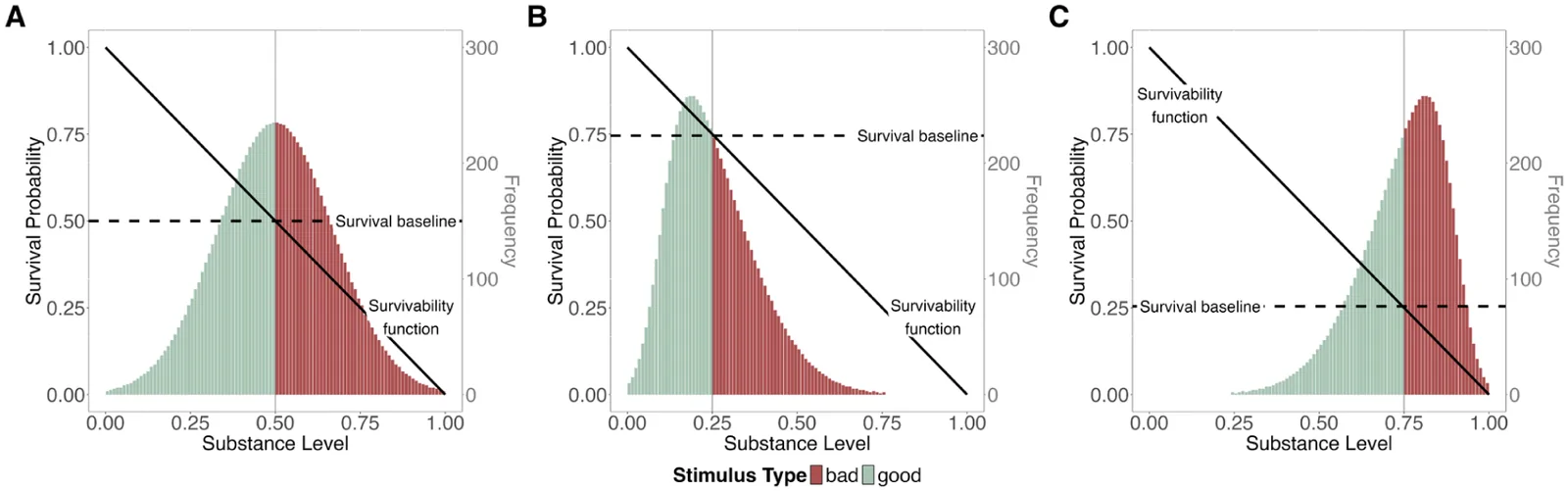
Survival probability and valence. *Note.* Valence (good vs. bad) is displayed as a function of substance level S=s , survivability function l(s) , and survival baseline $P(L|\bar{C})$ . Panel A shows normally distributed substance levels with an abolsute survival baseline of $P\left(L|\bar{C}\right)=0.5$ . Panel B shows right-skewed substance levels with a relative survival baseline, and Panel C shows left-skewed substance levels with a relative survival baseline.

Notably, defining good and bad purely in terms of the outcome of survival is a narrow understanding of the concepts and might contradict how most people use the terms in daily life. In everyday language, good and bad mostly refer to subjective evaluations of stimuli. Moreover, stimuli that are considered good or bad might be beneficial or harmful in terms of multiple different outcomes that sometimes contradict each other: A cigarette that has negative health impacts might still evoke pleasure. In contrast, we define good and bad as objectively existing properties of a stimulus, independent of any subjective evaluation, and assume that subjective evaluations correspond to the objective valence of a stimulus. This simplification is necessary to maintain sufficient parsimony to clearly delineate the theoretical ideas, but we will reflect on it in the discussion.

Our approach further implies that the meaning of good and bad depends on how we define the survival baseline. If we assume an absolute survival baseline, good and bad are stable labels, independent of any context and expectancies. In many modern living conditions, the vast majority of stimuli would likely be good. Even unpleasant temperatures would be labeled as good if they do not pose a serious danger to life. In contrast, if we assume the survival baseline to equal the expected survivability of all stimuli (relative survival baseline), the terms are entirely context-dependent. The same stimulus may be good in one environment, but bad in another. For instance, a moderately aggressive dog may be bad in a park with mostly peaceful dogs (as in Figure 2B), but good in a park in which most dogs are extremely aggressive (as in Figure 2C). This idea of a flexible evaluative neutral point can be traced, for instance, to Parducci’s (1965) range-frequency model.

### A Priori Survival Probability Without Processing

Environments can be more or less dangerous to perceivers, which is reflected in the *a priori probability of surviving a stimulus encounter* within a given environment. This becomes important when we introduce *processing mechanisms* ϕ later: Perceivers may process stimuli, and the more dangerous the environment, the more important such processing becomes.

We have established that environments differ in the substance distribution of their stimuli, their survivability function, and their survival baseline. To determine the a priori survival probability within an environment without any processing $P(L|\phi_{0})$ , we need to introduce another parameter, the probability P(C) that a stimulus makes contact in a given environment. Imagine a park where dogs must be kept on a leash versus one where dogs can roam freely. The probability that a dog will approach and make contact differs substantially between these environments. Consequently, the perceiver’s probability of being bitten by an aggressive dog or accompanied by a peaceful dog varies. We make the simplifying assumption that this *probability of contact* is constant across stimuli.

Jointly, these properties determine the a priori survival probability as follows:

(2) $$P(L|\phi_{0})=P(C)\times P(L|C)+(1-P(C))\times P(L|\bar{C})$$

The first term represents the case of getting in contact, and the second the case of non-contact. In essence, the a priori survival probability is determined by the expected survival probability when contact occurs, weighted by the probability of contact, and the survival probability without contact, weighted by the probability of non-contact. Remember that the expected survival probability when having contact with a stimulus follows from the survivability function in combination with the substance level distribution.

To later examine the differential survival advantage of processing good and bad stimuli, we must also distinguish their respective impact on the a priori survival probability. This impact depends on the likelihood of encountering good and bad stimuli. Our categorization yields two sets of stimuli, G and $\bar{G}$ . P(G) denotes the *proportion of good stimuli* G within all stimuli N . In a hospitable environment, P(G) exceeds .5; in a hostile one, it is below .5. If we further assume that every stimulus is either good or bad (i.e., no neutral stimuli), then the probability of encountering a bad stimulus is 1−P(G) .

To compare good and bad stimuli, we differentiate the average survival probabilities associated with contact and non-contact for each type, as illustrated in Figure 3. Formula 2 can then be expanded as follows:

(3) $$\begin{array}{l} P(L|\phi_{0})=P(G)\times (P(C)\times P(L|G,C)\\ \;\;\;\;\;\;\;\;\;\;\;\;\;\;\;\;\;\;\;\;\;\;\;\;\;\;\;\;\;\;\;\;\;+(1-P(C))\times P(L|G,\bar{C}))\\ \;\;\;\;\;\;\;\;\;\;\;\;\;\;\;\;\;\;\;\;\;+(1-P(G))\times (P(C)\times P(L|\bar{G},C)\\ \;\;\;\;\;\;\;\;\;\;\;\;\;\;\;\;\;\;\;\;\;\;\;\;\;\;\;\;\;\;\;\;\;+(1-P(C))\times P(L|\bar{G},\bar{C})) \end{array}$$

**Figure 3.**
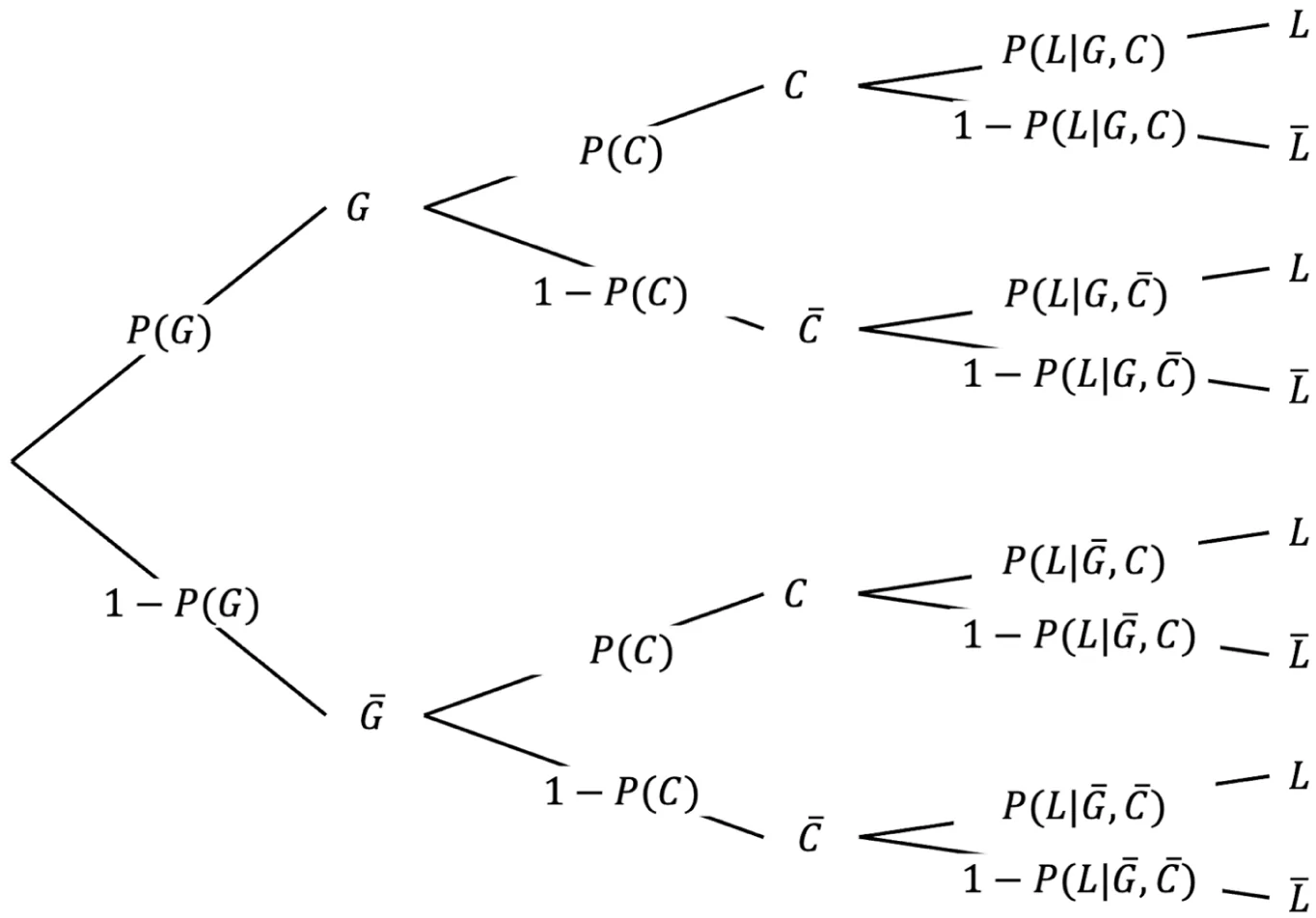
A priori probability of survival. *Note.* Probabilities of survival P(L) , conditional on the probability that a stimulus is good P(G) and the a priori probability of contact P(C) .

The survival probability of non-contact (i.e., the survival baseline) is independent of stimulus properties and thus identical for good and bad stimuli (i.e., $P(L|\bar{C},G)=P(L|\bar{C},\bar{G})$ ). We nevertheless retain both parameters separately to facilitate the later incorporation of differential processing. Note that Formulas 2 and 3 are identical in their outcomes.

### Processing

Now that we have established all key parameters of the environment, we no longer treat perceivers as passive. Instead, they may take action to make sure they actively approach stimuli that benefit their survival and avoid those that do not. For simplicity, we assume that these responses are always successful and enabled with perfect accuracy by the perceivers’ ability to process the relevant properties of the stimuli they seek to approach or avoid. When the perceiver in our example recognizes how aggressive a dog is, they can either avoid contact with it or, if the dog is calm, choose to approach. Thereby, processing effort can substantially enhance the perceiver’s survival probability.

Thus, in addition to the a priori probability that an encountered stimulus makes contact and the probability that it is good, survival now also depends on the probability that good and bad stimuli receive processing effort ( P(E|G) and $P(E|\bar{G})$ , respectively, with E denoting processing *effort*). This is depicted in Formula 4:

(4) $$\begin{array}{l} P(L|\phi )=\;P(G)\times [P(E|G)\times P(L|G,C)\\ \;\;\;\;\;\;\;\;\;\;\;\;\;\;\;\;\;\;\;\;\;\;\;\;\;\;\;\;\;\;\;\;\;\;\;\;\;\;\;\;\;+(1-P(E|G))\\ \;\;\;\;\;\;\;\;\;\;\;\;\;\;\;\;\;\;\;\;\;\;\;\;\;\;\;\;\;\;\;\;\;\;\;\;\;\;\;\;\;\times (P(C)\times P(L|G,C)\\ \;\;\;\;\;\;\;\;\;\;\;\;\;\;\;\;\;\;\;\;\;\;\;\;\;\;\;\;\;\;\;\;\;\;\;\;\;\;\;\;\;\;\;\;\;\;\;\;+(1-P(C))\times P(L|G,\bar{C}))]\\ \;\;\;\;\;\;\;\;\;\;\;\;\;\;\;\;\;\;\;\;\;+(1-P(G))\times [P(E|\bar{G})\times P(L|\bar{G},\bar{C})\\ \;\;\;\;\;\;\;\;\;\;\;\;\;\;\;\;\;\;\;\;\;\;\;\;\;\;\;\;\;\;\;\;\;\;\;\;\;\;\;\;\;\;+(1-P(E|\bar{G}))\;\times (P(C))\;\;\;\;\;\;\;\;\;\;\;\;\;\;\;\\ \;\;\;\;\;\;\;\;\;\;\;\;\;\;\;\;\;\;\;\;\;\;\;\;\;\;\;\;\;\;\;\;\;\;\;\;\;\;\;\;\;\;\;\;\;\;\;\;\;\;\;\;\;\;\;\times P(L|\bar{G},C)\\ \;\;\;\;\;\;\;\;\;\;\;\;\;\;\;\;\;\;\;\;\;\;\;\;\;\;\;\;\;\;\;\;\;\;\;\;\;\;\;\;\;\;\;\;\;\;\;\;\;+(1-P(C))\;\;\times P(L|\bar{G},\bar{C})] \end{array}$$

The basic structure of the formula remains the same as in Formula 3, but now the paths for good and bad stimuli branch to include cases where processing effort is directed toward either type, as illustrated in Figure 4.

**Figure 4.**
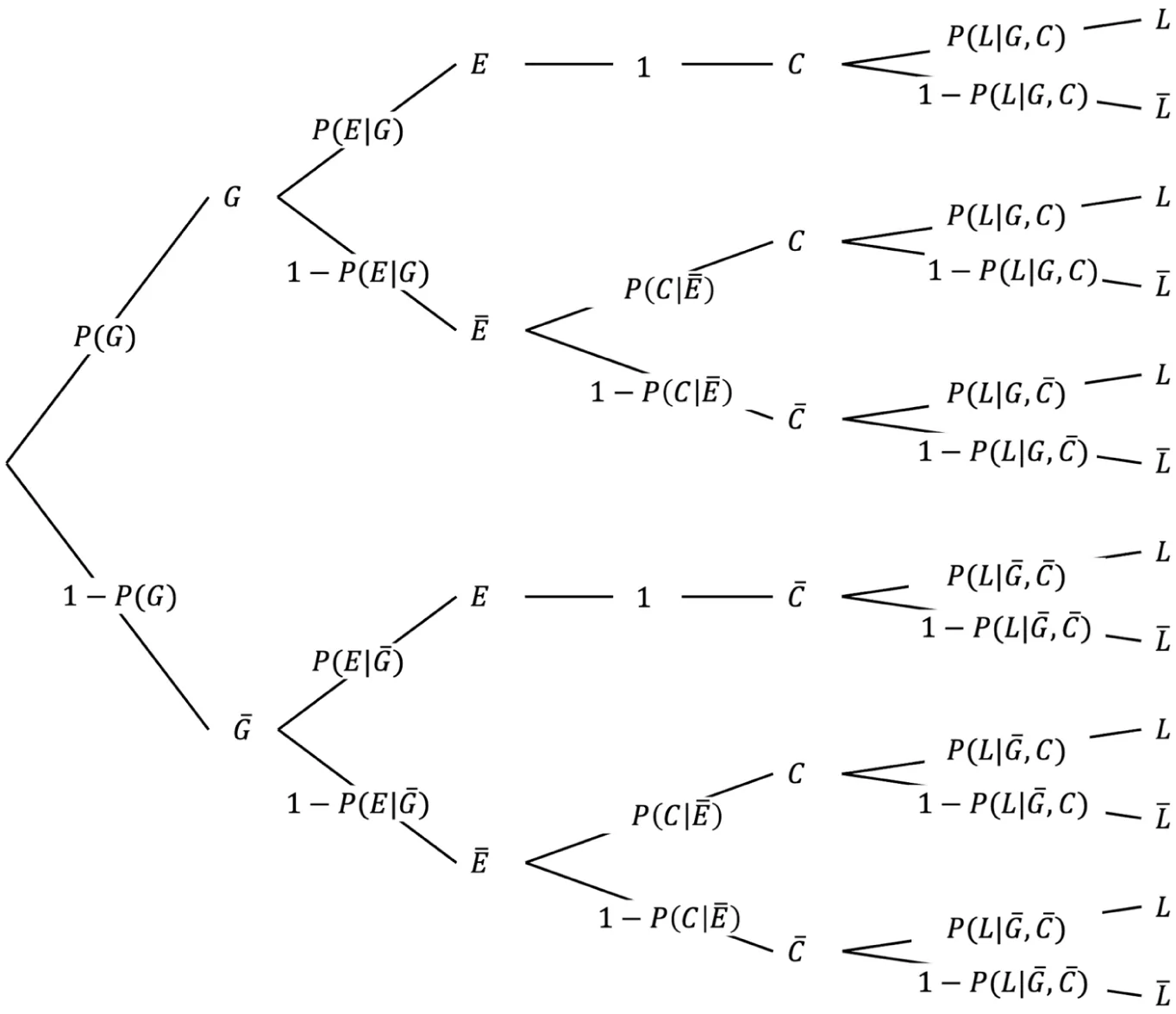
Survival probability with processing effort. *Note.* Probabilities of survival P(L) , conditional on the probability that a stimulus is good P(G) , the a priori probability of contact P(C) , and the probability of a stimulus to receive processing effort P(E|G) or $P(E|\bar{G})$ .

When no processing effort is applied, the survival probability depends on the likelihood that stimuli make contact or stay away by default, as well as the corresponding survival probabilities for these two cases, just as described in Formula 3. In contrast, when good stimuli receive processing effort, contact is guaranteed. Consequently, to the extent that processing effort is directed toward good stimuli, survival probability reflects the average survivability of good stimuli. When bad stimuli are given processing effort, they are successfully avoided so that no contact occurs. Thus, to the extent that processing effort targets bad stimuli, survival probability reflects the survival baseline.

If all stimuli were perfectly processed, all good stimuli would be contacted (after approaching the ones that would otherwise be missed) and all bad stimuli would be successfully avoided (after getting out of the way of the ones that would previously have hit). However, in a world with limited processing capacities, one type of stimulus may more likely receive processing effort while the other is neglected. Which kind of stimulus receives how much processing effort depends on the perceiver’s *processing mechanism* ϕ . We make the simplifying assumption that good or bad stimuli either receive full processing effort or none at all (i.e., the valence-specific probability of processing effort is binary: zero or one). Assuming a valence-driven processing mechanism, bad stimuli would receive effort while good ones would be neglected, resulting in the pattern $P\left(E|G\right)=0;\;P\left(E|\bar{G}\right)=1$ . Assuming instead that processing effort depends primarily on the distinctiveness of stimuli, as postulated by the distinctiveness-driven approach, implies more complex patterns, as we will show in the specification section.

Depending on environmental parameters (the proportion of good stimuli P(G) , the contact probability P(C) , the specific survivability values, and the survival baseline $P(L|\bar{C})$ , different processing mechanisms exert differential effects on the overall survival probability. Formula 4 allows comparing how different processing mechanisms influence survival in all kinds of environments.

#### Adaptive Advantage

Processing comes with an *adaptive advantage*, which we define as the gain in survival probability that a processing mechanism offers, given the characteristics of an environment. Adaptive advantage AA(ϕ) is thus the difference between the a priori survival probability without processing and the survival probability given a certain processing mechanism (Formula 5). Depending on the specific survivabilities of good and bad stimuli, the survival baseline, the proportion of good stimuli, and the probability of contact, the adaptive advantage associated with a given processing mechanism varies, as we will see when later specifying the valence-driven explanations.

(5) $$AA(\phi )=P(L|\phi_{0})-P(L|\phi )$$

### Multidimensional Stimuli

To later account for theoretical ideas on how the valence of multiple substance dimensions of the same stimulus is aggregated, we finally assume that more than one substance dimension may be relevant to the perceiver, such as a dog’s level of aggressiveness and the softness of its coat (note again that we refer to the non-verbal quality of the coat that makes it feel soft, not the subjective impression). We assume that each single dimension, denoted as ρ , has its own survivability function l(s) and substance level distribution $f_{S}(s)$ .

There are two competing ideas on how the survivability, and therefore the valence, of multiple dimensions might be connected: One may assume that all the individual survivability values of the different substance dimensions of a stimulus are simply averaged, which is illustrated for two substance dimensions in Figures 5A (linear survivability function) and 5C (concave survivability function). Both the valence-driven and distinctiveness-driven approaches, however, assume an aggregation by minimum, as shown in Figures 5B (linear survivability function) and 5D (curvilinear survivability function), and as defined in Formula 6:

(6) $$P\left(L|C,S=s\right)=\underset{\rho }{\min}P\;\;\left(\left(L|C,S_{\rho }=s_{\rho }\right)\right)$$

**Figure 5.**
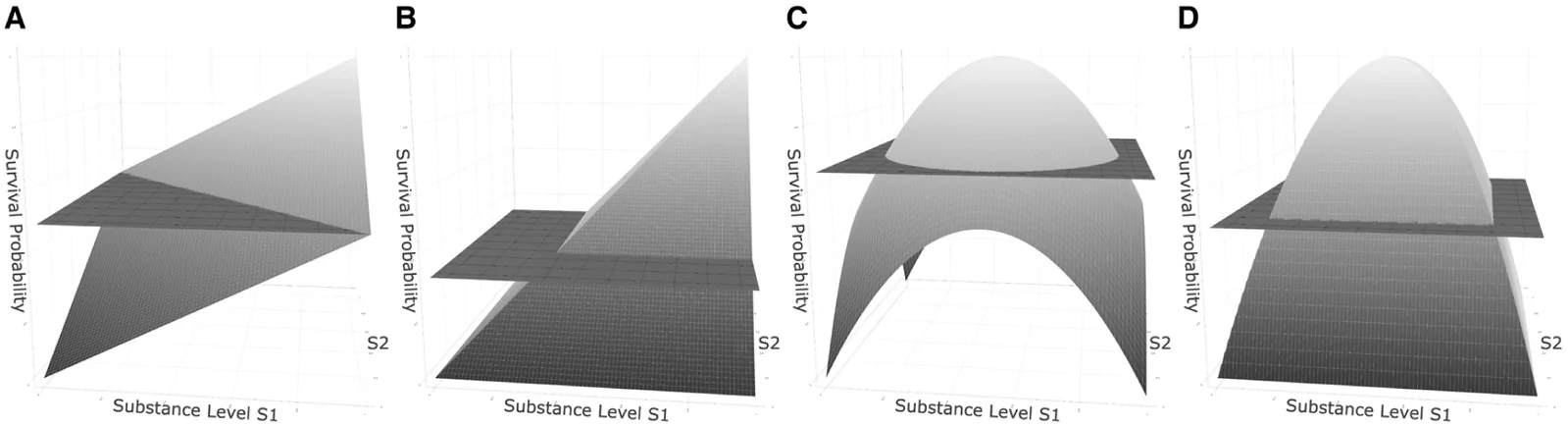
Survivability functions and survival baseline of two-dimensional stimuli. *Note.* Response surface for two substance dimensions (S1 and S2) whose survivabilities are aggregated either by average or by minimum. The dark-gray plane reflects the survival baseline; here, a relative baseline was chosen. The single-dimension survivability functions are identical to those in Figure 1. Substance distributions are ignored. Panels: (A) linear survivability function, aggregation by average; (B) linear survivability function, aggregation by minimum; (C) concave survivability function, aggregation by average; (D) concave survivability function, aggregation by minimum. Substance levels are normalized to range from 0 to 1.

Note that *S* = *s* now denotes the configuration of substance levels across dimensions, and *S*ρ = *s*ρ the level in a particular dimension.

This reflects the idea that in order to survive contact with a stimulus, one must survive contact with every characteristic of that stimulus. As soon as a stimulus is deadly in one dimension, the entire stimulus becomes deadly. It does not matter if the dog’s soft coat is good when its aggressiveness level is bad. Such an aggregation by minimum results in lower overall survivability values (compared to aggregation by average). In other words, it makes survival less likely on average.

Just as with unidimensional stimuli, the survivability function translates to the overall valence of a stimulus when being compared to the survival baseline $P(L|\bar{C})$ , that is, the survival probability when not getting in contact with a stimulus. Because the survival baseline is independent of substance, it is the same for the multiple substance dimensions. Note that, for multidimensional stimuli as well, the relative survival baseline adapts to the expected survivability and thus decreases when assuming conjunction by minimum, as depicted in Figure 5.

Of course, in reality, things are far more complex. For example, many substance dimensions may not have their own stable survivability functions but interact with other dimensions: An aggressive dog might not be dangerous per se, but only if it also has sharp teeth or carries a disease. We ignore such interactions here for simplicity.

### Summary

This is the complete model. While it might seem a bit overwhelming at first glance, there are in fact only five core parameters that determine everything else: the contact probability P(C) , the substance level distribution $f_{S}(s)$ , the survivability function l(s) , the survival baseline $P(L|\bar{C})$ , and the processing mechanism ϕ . From these variables follow the valence of a stimulus V , the proportion of good stimuli P(G) , the a priori survival probability without processing $P(L|\phi_{0})$ , the survival probability with a certain processing mechanism P(L|ϕ) , and the adaptive advantage of that processing mechanism AA(ϕ) .

## Reviewing the Two Approaches

On this basis, we can analyze the two major narrative approaches to valence asymmetries and specify their core concepts. The most important specification has already been made: clearly distinguishing good from bad stimuli. To examine the remaining concepts, we will start with the valence-driven approach, before turning to the alternative distinctiveness-driven approach.

Most fundamentally, the two approaches differ in the processing mechanisms they assume. They also differ in the environmental aspects with which they explain either the emergence of their assumed processing mechanism (in the valence-driven approach) or how the processing mechanism produces valence asymmetries (in the distinctiveness-driven approach).

### Valence-Driven Explanations

In the valence-driven approach, valence asymmetries emerge as a direct function of *valence-driven processing*; that is, the assumption that processing effort is inherently directed toward bad stimuli. Such selective, valence-driven processing is assumed to have emerged because it was adaptive to focus one’s processing effort on bad stimuli in ancient times, as these stimuli tended to be more *potent* than good ones (Rozin & Royzman, 2001). Other arguments for an adaptive advantage of this processing strategy follow from the so-called *asymmetry of life and death* and the *Chain Principle* (Baumeister et al., 2001). In the following, we specify valence-driven processing, the adaptive advantage of a certain processing mechanism, the potency of good and bad stimuli, the asymmetry of life and death, and the Chain Principle in terms of our model.

#### Valence-Driven Processing

The tenet is that processing effort directly depends on the valence of the encountered stimuli, independent of other characteristics or their current distribution in the environment. Processing bad stimuli is assumed to be prioritized over processing good stimuli, irrespective of anything else. Note that this idea presupposes that the perceiver determines the valence of the stimuli at the very initial stage of an encounter. The processing we are interested in only concerns analyses of additional stimulus features beyond valence (e.g., features that must be processed to enable an adaptive response). This dependence on valence is explained by a historical link between the potency and valence of stimuli, with bad stimuli being more potent, making it adaptive to focus one’s processing effort on them.

#### Adaptive Advantage

We specify the adaptive advantage of a processing mechanism AA(ϕ) as the difference between the a priori survival probability and the survival probability associated with processing a stimulus. This difference depends on the average survivability of good and bad stimuli, the survival baseline, the probability that encountered stimuli are good, and the probability that stimuli make contact by default.

While an adaptive advantage of certain processing mechanisms may influence how people process stimuli in past and present environments alike, in Baumeister et al.’s (2001) reasoning, it is primarily tied to phylogenesis. Processing mechanisms that have enhanced survival over a long period of time were passed down across generations, whereas maladaptive processing mechanisms gradually disappeared because perceivers using them did not survive long enough to pass along their genes. The assumption that the processing mechanism with the highest adaptive advantage through the course of evolution turned out to prioritize bad over good stimuli implies that processing bad stimuli rather than good ones increased the chances of surviving a stimulus encounter in ancestral environments more than alternative processing mechanisms.

We acknowledge that the most relevant variable for phylogenetic processes is actually not survival but procreation. Nevertheless, we use survival as a more parsimonious and easier-to-operationalize proxy for procreation, in line with Baumeister et al. (2001) and Rozin and Royzman (2001). We revisit the issue in the discussion.

#### Differential Potency

Potency is a frequently used construct in evolutionary approaches (Rozin & Royzman, 2001). Here, we model the potency of a stimulus as the impact that contact with that stimulus has on survival, which translates into the absolute value of valence. Like valence, the potency of a stimulus depends on the survivability function and the survival baseline. Good and bad stimuli may differ in their average potency, reflecting the idea that “negative events are more threatening than are positive events beneficial” (Rozin & Royzman, 2001, p. 314).

We define the absolute value of the average valence (i.e., potency) of all good stimuli as $Pot\left(G\right)=|P(L|C,G)-P(L|\bar{C})|$ , and the absolute value of the average valence (i.e., potency) of all bad stimuli as $Pot\left(\bar{G}\right)=|P(L|C,\bar{G})-P(L|\bar{C})|$ . When comparing the potency of good and bad stimuli, differential potency can be defined as the difference between these two average potencies: $dPot=Pot\left(G\right)-Pot\left(\bar{G}\right)$ . If this difference is positive, then good stimuli are more potent than bad ones (*positive differential potency*), and if it is negative, the opposite is true (*negative differential potency*).

Notably, differential potency is a characteristic of the given environment and may vary across contexts. It depends on the interplay between the shape and position of the survivability function, the survival baseline, and substance level distribution, as illustrated in Figure 6.

**Figure 6.**
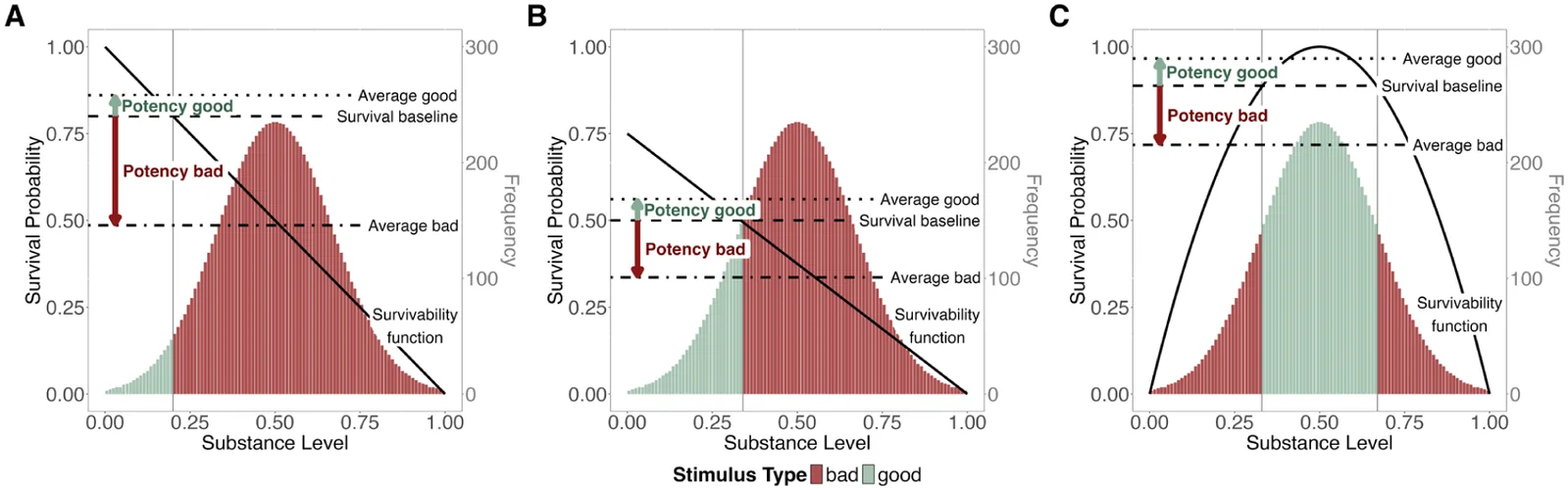
Differential potency of good and bad stimuli. *Note.* All panels show instances of negative differential potency. Panels: (A) Shows an increased absolute survival baseline of $P\left(L|\bar{C}\right)=.8$ . (B) Shows an absolute survival baseline of $P\left(L|\bar{C}\right)=.5$ and a compressed survivability function. (C) Shows a relative survival baseline and a concave survivability function.

When assuming an absolute survival baseline, a higher potency of bad compared to good stimuli may occur for three reasons: (a) the threshold from bad to good is very high (high survival baseline) so that the survivability of good stimuli can only slightly deviate from this high threshold while the survivability of bad stimuli has much larger room for deviation (Figure 6A), (b) the survivability of substance levels is rather low (compressed survivability function) so that the survivability of good stimuli is restricted (Figure 6B), or (c) the substance distribution has a particular shape (e.g., skewed to the positive pole; Figure 1A in the online supplement). More technically speaking, the potency of bad stimuli increases relative to that of good stimuli when the survival baseline moves upward or the average survivabilities of good and bad stimuli move downward.

When assuming a relative survival baseline, it takes a curved survivability function that roughly mirrors the distribution of substance levels to produce negative differential potency (Figure 6C). Otherwise, the survival baseline would shift upward or downward along with the average survivability, thereby diminishing any difference in potency (Figure 1B in the online supplement).

This reveals an important implicit assumption: Differential potency only occurs and can therefore only result in an adaptive advantage for valence-driven processing when assuming an absolute survival baseline or a very specific environmental configuration. Notably, in some environments, the pattern may be even reversed so that good stimuli are more potent than bad ones (e.g., when assuming an absolute survival baseline and a positively skewed substance distribution, Figure 2 in the Online Supplement), which will be relevant for the empirical setup that we propose later on.

In the case of differential potency, it is indeed more adaptive to process the more potent type of stimulus, as illustrated in Figure 7, which is face-valid given our definition of potency. Remember that by expending processing effort E , the perceiver switches from having contact with bad stimuli to avoiding them (no contact), and from missing good stimuli (no contact) to approaching them (contact). The greater the difference in survival probability between contact and non-contact (i.e., potency) for a certain type of stimulus, the greater the adaptive advantage of processing that stimulus. Thus, if bad stimuli are more potent, processing them is more adaptive, but if good stimuli are more potent, the reverse holds.

**Figure 7.**
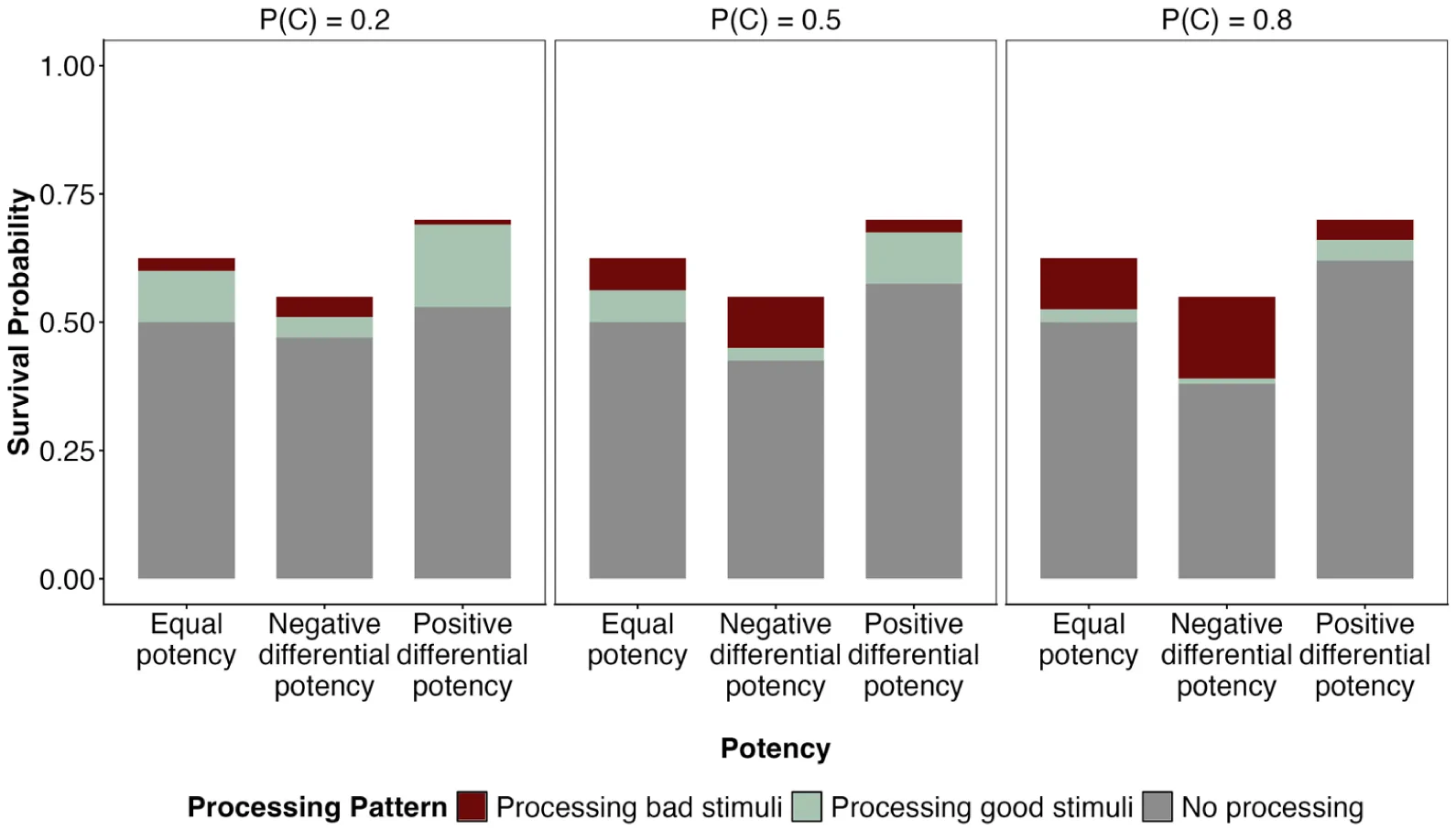
Potency and processing advantage. *Note.* Survival probability without processing and adaptive advantages of processing good and bad stimuli, assuming an absolute survival baseline of $P\left(L|\bar{C}\right)=.5$ and a proportion of good stimuli of P(G)=.5 . Contact probability P(C) varies from low Panel (A) to high Panel (C). Both adaptive advantages would add up if both types of stimuli were processed. Different assumptions on potency are represented by the three bars in each panel, based on the following specifications: left = equal potency of good and bad stimuli model, $P\left(L|C,\bar{G}\right)=.25,$ P(L|C,G)=.75 ; center = negative differential potency, $P\left(L|C,\bar{G}\right)=.1,$ P(L|C,G)=.6 ; right = positive differential potency, $P\left(L|C,\bar{G}\right)=.4$ , P(L|C,G)=.9 .

Beyond that, the effect of potency also interacts with the probability of contact P(C) and the proportion of good stimuli P(G) . Especially fascinating is the effect of the probability of contact, which has been completely overlooked in previous accounts. Think about the dog example again: The more likely it is that a dog makes contact, the more important it becomes to identify aggressive dogs to be able to avoid them. Conversely, the less likely it is that a dog makes contact, the less critical it becomes to watch out for aggressive dogs, and the more advantageous it becomes to seek out calm dogs to approach them and remain safe in their company. This pattern becomes apparent in Figure 7, where the adaptive advantage of processing bad stimuli increases from the left to the right panel, whereas the adaptive advantage of processing good stimuli decreases. The effect persists even when bad stimuli are more potent than good ones (middle bar in all panels of Figure 7).

##### Chain Principle and Potency

In the narrative literature on valence asymmetries, differential potency is not only described as a characteristic of stimuli per se but also explained by the *Chain Principle* (Weinberg, 1975), the idea that a system is only as strong as its weakest component (Baumeister et al., 2001). This necessarily implies that a stimulus must possess more than one characteristic relevant to the perceiver’s survival.

Using our formal model, we can incorporate this idea by aggregating the survivabilities of multiple substance dimensions by their minimum (see *Multidimensional Stimuli*) so that the overall valence of a stimulus is determined by the lowest survivability in any of its dimensions. This means that stimuli can only be good when all their characteristics are good, rather than being merely good on average. We will later show that this reflects the same mechanism that the distinctiveness-driven approach introduces as the *Anna Karenina Principle* (Diamond, 1997; Moore, 2001). Note that aggregation by minimum is just an assumption that may reflect reality more or less accurately and may be proven wrong empirically.

The impact of how the survivabilities of multiple substance dimensions are aggregated on potency depends entirely on the type of survival baseline assumed. With an absolute survival baseline, aggregation by minimum indeed results in a higher potency of bad stimuli and thus an adaptive advantage of focusing one’s processing on bad stimuli, as illustrated in Figure 8A and B: When assuming aggregation by minimum (Figure 8B) instead of average (Figure 8A), good things become less good, bad things become worse and some things that might have been good before even become bad. This reduces the difference between the average survivability of good stimuli and the survival baseline (compare the good and the neutral plane) while enhancing the difference between the average survivability of bad stimuli and the survival baseline (compare the bad and the neutral plane). This negative differential potency may then constitute an adaptive advantage in just the same way as for unidimensional stimuli.

**Figure 8.**
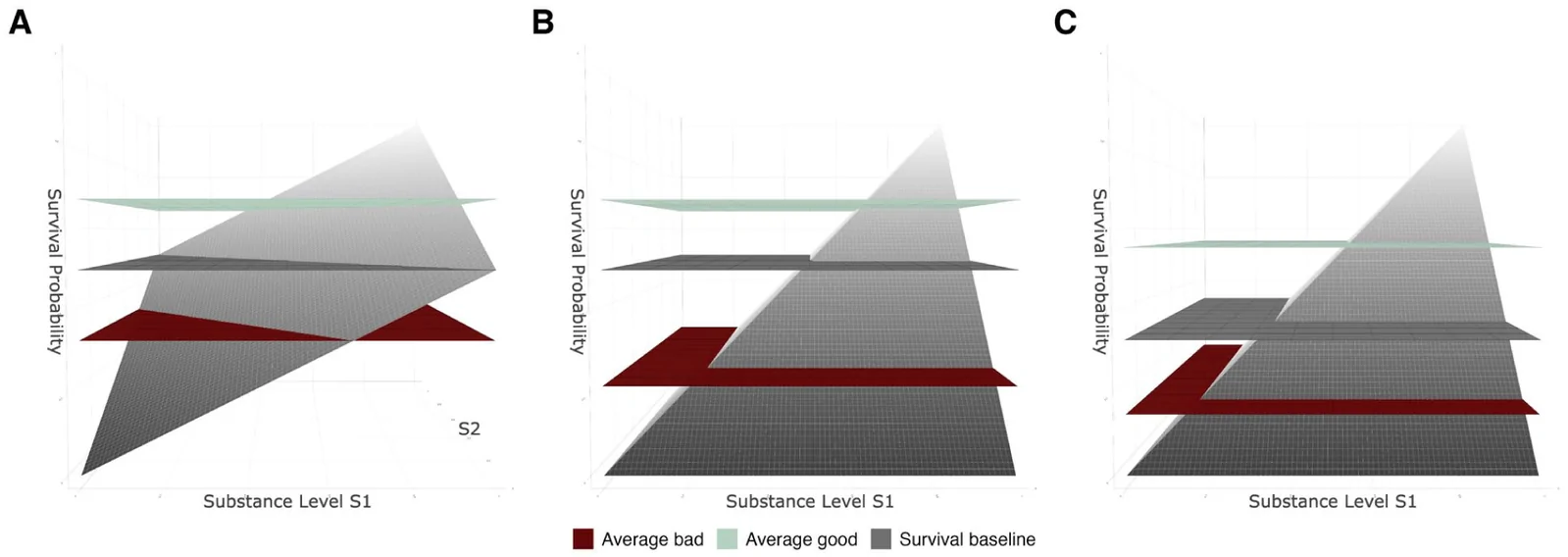
Effects of type of aggregation on the average potency of good and bad stimuli. *Note.* Impact of aggregation rule across two substance dimensions (S1, S2) on the average potency of good and bad stimuli. Planes for average survivabilities of good and bad stimuli, P(L|C,G) and $P\left(L|C,\bar{G}\right),$ were added to Figure 5A and B. Only linear survivability functions are displayed. Panels: (A) aggregation by average (absolute survival baseline of *P*(*L*|*C* with horizontal bar) = 0.5 and relative survival baseline are identical), (B) aggregation by minimum, absolute survival baseline of *P*(*L*|*C* with horizontal bar)=0.5, and (C) aggregation by minimum, relative survival baseline. Substance levels are normalized to range from 0 to 1.

However, for a relative survival baseline, things are again complex. Because the survival baseline shifts downward along with the overall survivability of the stimuli, the relative potency of good and bad stimuli becomes more balanced or even reversed. This can be seen by comparing the good and the bad planes with the survival baseline in Figure 8C.

Overall, an aggregation by minimum (i.e., the chain principle) may only clearly explain differential potency and an adaptive advantage of processing bad stimuli when assuming an absolute threshold between good and bad. When assuming that the threshold from bad to good is relative to the environment, an aggregation by minimum results in a lower threshold, so that poorly survivable stimuli are considered normal, and it is the positive stimuli that stand out (i.e., are more potent) for their relatively high survivability. Note that the interplay between aggregation rule and survival baseline might be influenced by the substance level distribution which we ignored for now.

#### Asymmetry of Life and Death

Another factor proposed to underlie an adaptive advantage of valence-based processing, favoring bad stimuli, is the *asymmetry of life and death* (Baumeister et al., 2001; Rozin & Royzman, 2001): To survive multiple consecutive stimulus encounters, an organism must survive each one of them. Because death must be avoided in all instances, bad stimuli are assumed to warrant more processing effort than good ones.

Formally, this asymmetry corresponds to the multiplicative accumulation of survival probabilities across several encounters: For n encounters, all survival probabilities are raised to the power of n , resulting in a cumulative survival probability without processing $P{\left(L|\phi_{0}\right)}^{n}$ and one with a certain processing mechanism $P{\left(L|\phi \right)}^{n}.$ Consistent with Baumeister et al. (2001), this formalization suggests that the probability of survival without processing declines with the number of encounters. In other words, as time passes and encounters accumulate, the probability of dying inevitably increases.

If a processing mechanism could guarantee survival with absolute certainty, its adaptive advantage would increase with each encounter. In this scenario, the survival probability without processing would steadily decline, while the probability with processing would remain constant at one. In most environments, however, no processing mechanism can fully ensure survival. If the environment is not optimal, even optimal processing cannot prevent death indefinitely. As soon as the survival probability with processing is below one, it too decreases as more stimuli are encountered. Initially, it may decline more slowly than the survival probability without processing, but over time, both trajectories converge toward zero. While processing still extends survival, its adaptive advantage gradually decreases, and death is ultimately inevitable.

Nevertheless, as outlined in the previous section, the processing mechanism that maximizes survival does not necessarily prioritize bad stimuli. In some environments, focusing on good stimuli may be more adaptive than focusing on bad ones. Mathematically, the processing mechanism yielding the highest survival probability remains superior across encounters—the relative differences between patterns may change, but their rank order does not. Hence, multiple encounters may amplify existing advantages of processing mechanisms but cannot generate entirely new processing preferences.

#### Conclusion

Our analysis showed that environmental factors and assumptions about survivability and valence play a crucial role in explaining how valence-driven processing may present an adaptive advantage.

Previous literature attributes this adaptive advantage to differential potency, the Chain Principle, and a multiplicative accumulation of survivabilities over multiple stimulus encounters (i.e., the asymmetry of life and death). Our analyses suggest that bad stimuli are only more potent and warrant more processing effort under certain assumptions. If we consider the survival baseline—the threshold between bad and good—to be independent of the environment, several factors may explain a higher potency of bad as compared to good stimuli: a high survival baseline, compressed survivability function, skewed substance level distribution, or aggregating several substance dimensions by their minimum. If we regard the survival baseline to be relative to the environment, only a curvilinear survivability function may result in a higher potency of bad stimuli.

The analyses also highlight that not only differential potency but also a high contact probability may explain an adaptive advantage of prioritizing processing effort for bad stimuli. Finally, the multiplicative accumulation of several stimulus encounters may amplify existing advantages of a processing mechanism for some time before they start to decline at some point.

Traditionally, the proposed mechanisms are linked to phylogenesis. Their presence and the resulting adaptive advantage in past generations are thought to have produced the observed processing patterns today. However, adaptivity need not be necessarily tied to phylogenesis; it may also influence processing during ontogenesis or be manipulated in experimental settings.

### Distinctiveness-Driven Approach

The distinctiveness-driven approach offers an alternative explanation of valence asymmetries. It assumes that, in typical environments, bad stimuli receive more processing effort than good stimuli, not because of their valence, but because they are more distinct—that is, less similar and less frequent than good stimuli. This is mainly explained with the so-called *Range Principle* (e.g., Alves et al., 2017; Unkelbach et al., 2020) and the *Anna Karenina Principle* (Diamond, 1997; Moore, 2001).

#### Distinctiveness-Driven Processing

The tenet is that processing effort is ultimately directed toward *distinct* stimuli. This view is in line with a more general Bayesian perspective on processing, such as the idea of the *predictive brain*: There is an expected value for most stimuli (e.g., the typical size of a human being), and incoming information triggers more processing effort the more it *deviates* from that prior (e.g., Clark, 2013; Shin & Niv, 2021).

Valence asymmetries may thus occur simply because good and bad stimuli happen to differ in distinctiveness. In more technical terms, a processing pattern that favors the processing of bad stimuli emerges because distinctiveness and valence are negatively related. When bad stimuli are more distinctive than good ones, they receive greater processing effort. Conversely, if *good* stimuli were more distinctive, they would attract more processing effort, and valence asymmetries would reverse (e.g., Alves et al., 2015; Sperlich & Unkelbach, 2025).

#### Differential Distinctiveness

As stated, both similarity and frequency are invoked as predictors in this approach. We subsume the opposites of both under the broader *distinctiveness* label, because their predicted effects are typically identical. Note that the relationships of similarity and frequency with distinctiveness are negative.

##### Similarity

*Similarity* is one of two key concepts in the distinctiveness-driven approach. The connection between similarity and distinctiveness is negative, such that more similar stimuli are less distinct. We specify the similarity of good stimuli Sim(G) and the similarity of bad stimuli $Sim\left(\bar{G}\right)$ as the average proximity of the substance levels of all good and bad stimuli to their valence-specific means.^1^ Differential similarity is the difference between these two valence-specific similarities: $dSim=Sim\left(G\right)-Sim\left(\bar{G}\right)$ . It may range between −1 and 1, with positive values indicating a greater similarity among good than among bad stimuli, and negative values indicating the opposite. Accordingly, bad stimuli are expected to be more distinct and thus receive more processing effort the more positive dSim is. Remember that we focus here on objective similarity, that is, actual differences in the substance levels of good versus bad stimuli, rather than the perceived similarity that is typically measured in empirical studies (e.g., Alves et al., 2016; Koch et al., 2016).

##### Frequency

Another aspect of distinctiveness is the *frequency* of stimuli. Good stimuli may not only be less distinctive than bad stimuli because they are more similar to one another, but also because they occur more frequently (Alves et al., 2017). We model the relative frequency of good and the relative frequency of bad stimuli with the P(G) and 1−P(G) parameters introduced in the model section. Differential frequency occurs when bad stimuli are more or less frequent than good stimuli. It is the difference between those two relative frequencies: dFreq=P(G)−(1−P(G)) . Positive values indicate a greater frequency of good and a greater rarity of bad stimuli, whereas negative values imply the reverse. We will later show that the assumed type of survival baseline may have a huge impact on the differential frequency of good and bad stimuli.

##### The Range Principle and Differential Distinctiveness

Differential frequency and similarity are both explained using the so-called *Range Principle* (Alves et al., 2017; Unkelbach et al., 2020). The Range Principle states that there typically exists one range of good substance levels that is framed by two ranges of bad substance levels. Our model determines the number and width of these ranges by the shape and position of the survivability function, combined with the assumed survival baseline, as shown in Figure 9.

**Figure 9.**
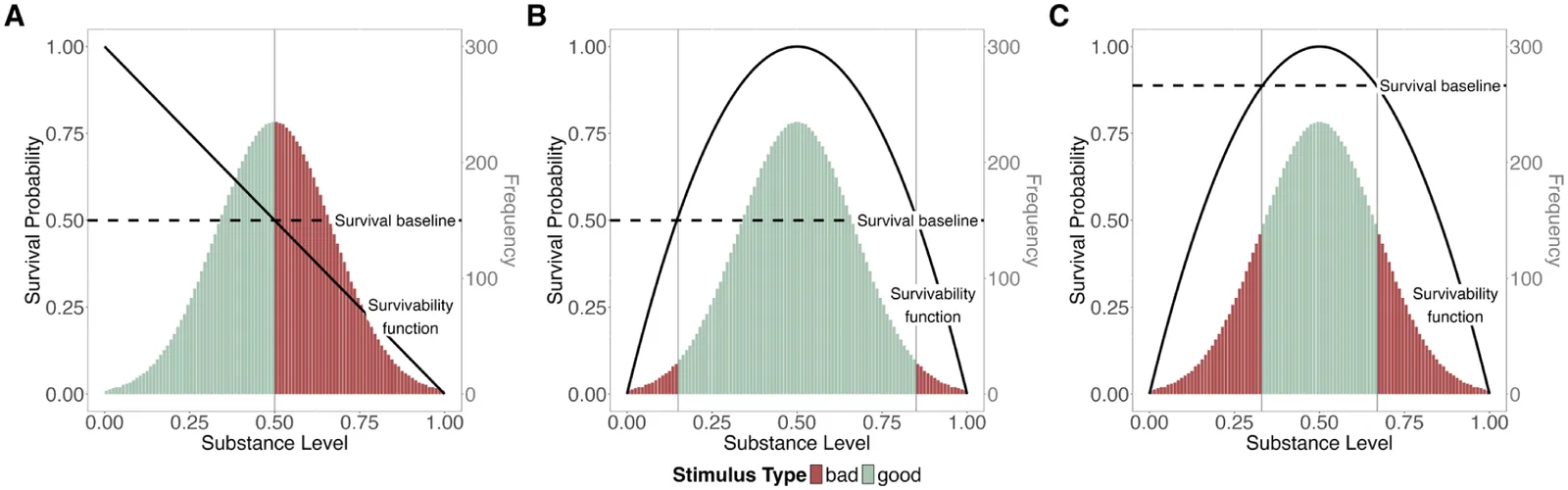
Dependence of range width on survivability function, survival baseline, and substance level distribution. *Note.* Panel (A) shows a linear survivability function with an absolute survival baseline of $P\left(L|\bar{C}\right)=.5$ . Panel (B) shows a concave survivability function with an absolute survival baseline of $P\left(L|\bar{C}\right)=.5$ . Panel (C) shows a concave survivability function with a relative survival baseline.

The most parsimonious assumption would be a linear survivability function, so that there is one desirable and one undesirable pole for every substance dimension, as depicted in Figure 9A. This corresponds to our example of a dog encounter: the more aggressive the dog, the lower the chances of survival. Such a linear survivability function results in two ranges, a bad and a good one, and the similarity and frequency of good and bad stimuli depend on the width of these ranges and the distribution of substance levels.

However, Unkelbach, Alves, and Koch (see Alves et al., 2017; Unkelbach et al., 2008, 2020) argue that for most substance variables (e.g., ambient temperature), there is not a linear but a *concave* (inversely *U*-shaped) relationship between substance and valence. Because we define valence in terms of survival probability, this would translate to a concave survivability function in our model. Such a concave function typically produces one positive range that is framed by two negative ranges, as shown in Figure 9B and C. The specific shape of the function and the position of the survival baseline can differ (absolute survival baseline in Figure 9B, relative survival baseline in Figure 9C), and therefore the ranges can be narrower or broader—but as long as the survivability function is concave, there remains one range of good stimuli framed by two ranges of bad stimuli.

In our model, a concave survivability function coupled with a normal distribution of substance levels results in differential similarity, independent of the assumed type of survival baseline or the width of the ranges. With an increasing breadth of the inner (good) range, the distance between the two outer (bad) ranges grows as well. For example, good temperatures are more similar, whereas bad ones are more distinct, because it can either be too cold or too hot. This holds both when the range of good temperatures is broad and when it is narrow.

The Range Principle also results in differential frequency. Assuming a concave survivability function and normally distributed substance levels leads to a higher frequency of good stimuli and, consequently, greater rarity of bad ones, even when a relative survival baseline is assumed, due to the steeper slope at the edges of the substance scale (see Figure 9C). Still, such a relative threshold between good and bad precludes the emergence of extreme differences in frequency.

Taken together, assuming distinctiveness-driven processing, a concave survivability function as implied by the Range Principle, and normally distributed substance levels, provides a plausible explanation for the observed negativity bias in processing effort, regardless of the assumed survival baseline. Just as in the valence-driven approach, however, the validity of these assumptions remains an empirical question.

##### Anna Karenina Principle and Similarity

Differential similarity is not only explained by the Range Principle but also by the *Anna Karenina Principle* (Diamond, 1997; Moore, 2001), which suggests that a stimulus can only be good when all of its characteristics are good. In consequence, good stimuli are argued to be more similar to one another than bad stimuli.

This idea translates to an *aggregation by minimum* across several characteristics of the same stimulus, as explained in the model description (see *Multidimensional Stimuli*): The overall survivability of a multidimensional stimulus is determined by the lowest survivability associated with any of its substance levels, rather than, for example, by the average of all individual survivabilities (*aggregation by average*). The Anna Karenina Principle is thus conceptually identical to the Chain Principle discussed within the valence-driven approach. While the valence-driven approach focuses on how such an aggregation by minimum affects potency, the distinctiveness-driven approach focuses on its relation to differential distinctiveness.

While Unkelbach, Alves, and Koch (2020) argued that the Anna Karenina principle is a direct consequence of the range principle, we now argue more specifically that the concave shape of survivability functions $l(s_{\rho })$ (implied by the Range Principle) and the way in which individual characteristics’ survivabilities are combined (e.g., by averaging vs. by minimum) are two independent factors, which influence the similarity of multidimensional stimuli in different ways.

Figure 10 illustrates how aggregation by minimum increases similarity among good stimuli, independent of the shape of the survivability functions. This applies not only when survivability functions are concave (Figure 10A vs. B) but also when they are linear (Figure 10C vs. D).

**Figure 10.**
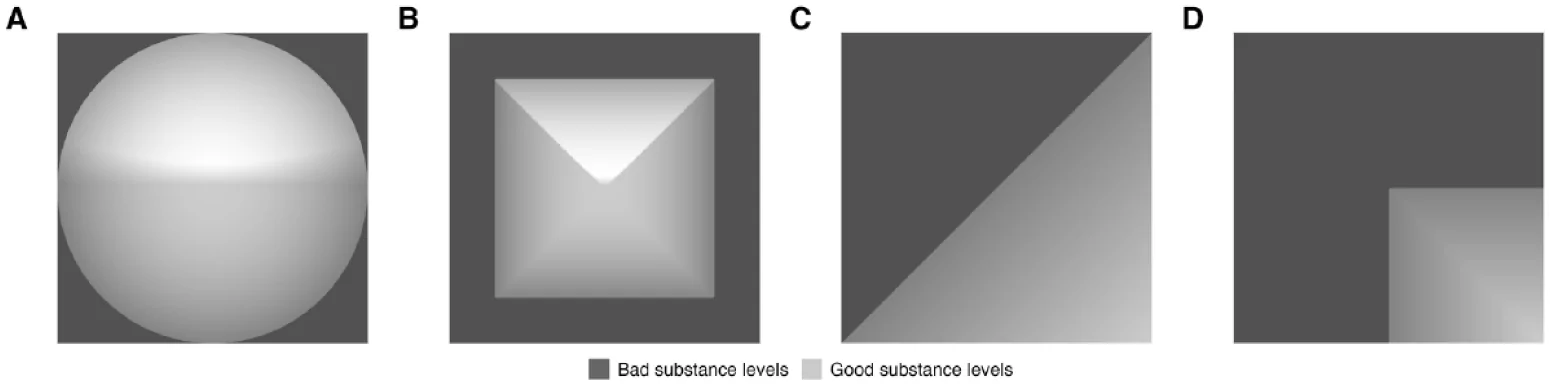
Similarity of good versus bad multidimensional stimuli. *Note.* Top-down view based on Figure 5, showing how the aggregation rule and survivability function affect which combinations of substance levels in two-dimensional stimuli are classified as good or bad. An absolute survival baseline of survivability function $P(L|\bar{C})=.5$ is assumed. Panels: (A) concave survivability function, aggregation by average; (B) concave survivability function, aggregation by minimum; (C) linear survivability function, aggregation by average; (D) linear survivability function, aggregation by minimum. The spatial distribution of the two different areas reflects the differential similarity of the two types of stimuli. For example, the substance levels of good and bad stimuli are evenly distributed and therefore equally similar in Panel C, whereas the substance levels of good stimuli are surrounded by bad ones and therefore more similar in Panel B.

Independent of that, concave survivability functions for all substance dimensions (i.e., the Range Principle) result in higher similarity among good and higher diversity among bad multidimensional stimuli than linear survivability functions regardless of the aggregation rule: Good stimuli whose multiple substance dimensions have concave survivability functions are also more similar than stimuli with linear survivability functions if substance dimensions are aggregated by average (Figure 10A vs. C), not only when they are aggregated by minimum (Figure 10B vs. D).

Overall, consistent with Unkelbach, Alves, and Koch (2020), our model implies that bad stimuli may even appear more distinct when looking at multiple substance dimensions at once. However, this is not necessarily due to an aggregation by minimum but may also directly follow from the concave survivability functions that are assumed in the Range Principle.

#### Conclusion

Our formalization gave us several new insights about distinctiveness-driven approaches: First, we could confirm that the range principle results in a higher distinctiveness of bad stimuli. This is very robustly the case in terms of a lower similarity of bad as compared to good stimuli. Concerning frequency, potential differences between good and bad stimuli are present with both types of survival baselines, but somewhat limited when assuming a relative survival baseline. Second, when regarding multidimensional stimuli, a higher similarity of good as compared to bad stimuli may result from two factors that are not necessarily linked: concave survivability functions in all substance dimensions and aggregation by minimum across the dimensions. In previous accounts (e.g., Unkelbach, Alves, & Koch, 2020), these two factors have been conflated.

Interestingly, when assuming concave survivability functions and normally distributed substance levels, bad stimuli are not only more distinct but also more potent (see Figure 6C). Thus, in such an environment (which Unkelbach, Alves, & Koch, 2020, assume to be quite common), not only are valence and distinctiveness confounded, but also potency and distinctiveness.

## A Framework for Comparative Experimentation

Beyond theoretical clarity, specifying the key concepts in both approaches enables the development of an empirical framework for comparative experimental tests. We first revisit the overarching argumentative structure to clarify which aspects of the two approaches exactly need to be compared. To rule out alternative explanations, we highlight other explanatory mechanisms beyond the ones proposed in the valence-driven and the distinctiveness-driven approach that are implied by our model and might also account for observed valence asymmetries. We then propose a possible experimental framework to compare these different explanations.

### Explanatory Mechanisms

Our specification of two theoretical approaches highlights different routes in explaining the empirically observed processing differences, each involving at least one assumption concerning the processing focus and at least one concerning past or present environments. For empirical research, both aspects must be jointly considered to enable comprehensive insights into the underlying causes of valence asymmetries.

Our framework assumes that the environment is characterized by substance-level distributions, survivability functions, and survival baselines. Ultimately, processing always happens on the substance level. The substance level is what a perceiver observes and uses for all subsequent processing and potential responding. The substance level of a stimulus may be more or less distinct depending on its deviation from the average substance level within a given environment. It may also have a specific valence and potency, depending on the relationship between the survivability function and the survival baseline.

The processing mechanism concerns the question of what exactly determines processing effort. The most parsimonious assumption would be that—for whatever reason—people prefer certain stimulus characteristics, for example, large size or saturated color. Those stimulus characteristics would then evoke the most processing effort. Almost as straightforward is the assumption that people focus their processing effort on distinctive stimuli. Distinctiveness, however, is only possible when comparing multiple stimuli, present or encountered in the past. The assumption that people expend processing effort based on the valence of a stimulus is more complex, because knowing the valence of a stimulus requires prior contact. Beyond that, our model suggests some alternative processing mechanisms: for instance, processing effort could be directly driven by the potency of a stimulus instead of its valence. Individuals may direct their processing effort to the stimuli that they have learned to be more potent, in positive as well as negative ways. Finally, even more complex cases are conceivable: individuals might use one feature as a proxy for another or show no fixed processing tendency at all, instead adjusting processing effort depending on what proves to be adaptive.

Within any given environment, distinctiveness, valence, and potency can be related in various ways. Valence asymmetries should emerge to the extent that the focus of the processing mechanism and valence are correlated within an environment.

In the distinctiveness-driven approach, the allocation of processing effort is assumed to be guided by stimulus distinctiveness. Valence asymmetries are expected to emerge when distinctiveness and valence are negatively correlated—that is, when negative stimuli tend to be more distinctive than positive ones.

In contrast, the valence-driven approach assumes that processing effort is directed toward bad stimuli, irrespective of their distinctiveness. This approach assumes that valence and potency have been linked in ancestral environments and may still be linked today. The tendency to prioritize the processing of negative stimuli is regarded as phylogenetically transferred and stable.

Upon closer examination, however, it appears unclear what type of processing focus is actually implied by verbal accounts of the valence-driven approach. Is processing effort directed at the valence of a stimulus or its potency? This translates to the question of whether valence asymmetries are observable across all environments, or only when valence and potency are positively linked. Another uncertainty is the role of phylogenesis in this regard. Does the assumed mechanism work better for specific substance features that have been historically associated with valence and/or potency, or can these associations be learned for any substance dimension? This is linked to the notion of preferanda (Zajonc, 1980): stimulus properties that have been phylogenetically learned to signal danger and thus attract processing attention independently of their current valence or potency.

By integrating environmental aspects, assumed processing mechanisms, and observable processing outcomes, our formal account suggests experimental designs that are capable of distinguishing between distinctiveness-driven, valence-driven, potency-driven, or more complex processing mechanisms. In the following, we outline an exemplary experimental setup to illustrate how our model can be employed to provide clarity.

### Experimental Setup

In typical environments, including most experimental settings, substance levels, valence, potency, and distinctiveness tend to be confounded in one way or another. As a result, it is often impossible to draw valid conclusions about processing mechanisms based on the observed patterns of processing effort. An informative experimental setup must disentangle the different explanatory factors by removing these confounds. Processing mechanisms may then be inferred from the interplay between environmental factors and observed response patterns to different stimuli. There have been similar approaches in the past (e.g., Fazio et al.’s (2004) BeanFest paradigm), but if our framework bears some truth, these experimental approaches were incomplete.

Let us thus propose an exemplary paradigm: Participants engage in a game designed to simulate an individual’s behavior in an abstract environment. The game resembles a classic early video game scenario in which the player controls a spaceship at the bottom of the screen, which can only move to the left or right. A survival counter tracks their remaining level of vitality. During the game, abstract objects fall from the top of the screen. Each newly introduced object is a new stimulus encounter, which may result in contact or no contact with the spaceship. The objects can either appear right above the spaceship so that they would get in contact automatically, or next to the spaceship.

Each object is assigned a valence, potency, and distinctiveness. This can be done by specifying the parameters of the formal model, which are all under the experimenter’s control. In the simplest version of the paradigm, the objects differ in only one substance dimension (e.g., their size). Determined by the substance level distribution, stimuli of different substance levels automatically differ in distinctiveness. Depending on the survivability function, each substance level also has its own survivability, which can only be determined by establishing contact with objects: contact with certain stimuli reduces vitality more than contact with other stimuli does. In addition, not having contact with objects will also affect vitality to some extent. This is the survival baseline. The baseline can be set to be absolute or relative to the average survivability of all stimuli in the game environment. In interplay with the survivability function, the position of the survival baseline determines the valence and potency of a stimulus. Compared with the survival baseline, some (“good”) stimuli should be contacted, and some (“bad”) stimuli should be avoided. Moreover, some stimuli are more potent than others. That is, contacting them or avoiding them will lead to smaller or greater losses of vitality than contacting or avoiding other stimuli will.

Participants can move the ship to enhance their chances of survival by approaching good stimuli and avoiding bad ones. The game ends when the survival counter reaches zero. The overall goal is to stay alive as long as possible.

This setup enables several different types of analyses, depending on the research question, and allows the incorporation of different dependent measures to explore the specific positivity and negativity biases that are currently subsumed as processing effort. By varying the environmental setup, experimenters can not only manipulate distinctiveness, valence, and potency but also their covariation to disentangle their influence on processing outcomes. One may also manipulate the default contact probability and thus disentangle potency from adaptivity by varying the tendency of objects to appear right above the spaceship (contact by default) or next to it (no contact by default). A comprehensive account would systematically vary the correlations between distinctiveness, valence, potency, and adaptivity, and compare the processing patterns that the different explanations predict in each of these environments with the observed patterns. This allows for a nuanced analysis of the environmental conditions and processing assumptions under which valence asymmetries can be reproduced. By identifying which predicted pattern most closely matches the data, inferences can be drawn about the processing mechanism that best explains participants’ behavior.

For example, the classic negativity advantage (i.e., the valence explanation) would be supported if participants recognize negative stimuli better in all possible environments (or recall or react correctly to them), independent of potency, contact frequency, or general survivability (i.e., hostile or benign playing environments). Not surprisingly, the distinctiveness explanation would be supported if participants recognized distinct stimuli better - even if this comes with an adaptive disadvantage. Neither of these explanations would be supported if participants just used any cue for what is adaptive to further process in their current environment without a clear preference for bad, distinct, or potent stimuli.

## Discussion

We aimed to advance the scientific discourse on valence asymmetries by translating two theoretical approaches from narrative accounts into formal terms and analyzing how their underlying logic may explain the pervasive negativity and positivity biases documented in empirical research. Based on the conceptual insights that we gained, we believe that this effort was largely successful.

### Key Insights

One of the major insights from our conceptual analyses was the importance of clearly defining what is meant by *good* and *bad*. Good or bad compared to what? This pertains directly to the relevance of the survival baseline $P(L|\bar{C})$ . Our analyses show that the choice of survival baseline determines which stimuli, based on their respective survivability, will be considered good or bad for the perceiver. This, in turn, crucially affects which processing mechanisms will be most adaptive. Our analyses imply that both reviewed approaches may have implicitly employed an absolute survival baseline. However, in terms of ecological validity, employing relative survival baselines (i.e., ones that reflect the average survival probabilities in the given environment) may actually be more reasonable. In hostile environments, approaching those stimuli with the highest survivability at contact will be adaptive, even if these survivabilities are low compared to some absolute standard. Future research into valence asymmetries needs to be more explicit about which type of baseline is assumed and why.

A second key insight was that researchers need to specify their assumptions not only about the assumed proportion of good stimuli P(G) but also about the a priori probability of getting in contact P(C) . In terms of processing necessity, it makes a difference whether remaining passive results in contact or non-contact with encountered stimuli. It seems that most valence asymmetry research, especially within the valence-driven tradition, has implicitly assumed a high contact base rate.

Comparing the two theoretical approaches, we found that their traditional labels are somewhat misleading, because *intrapsychic*, *phylogenetic*, *ecological*, and *ontogenetic* aspects may be at play in both explanatory strands. To more precisely capture the core difference between them, we therefore referred to the two perspectives as the *valence-driven* and the *distinctiveness-driven* approach.

Within the valence-driven approach, our analyses confirmed that a higher potency of bad stimuli indeed fosters an adaptive advantage for prioritizing bad stimuli in processing. However, such potency differences are much more likely to occur under specific environmental conditions: either an absolute survival baseline or a curved survivability function that roughly mirrors the distribution of substance levels. Notably, the latter is in line with the environmental assumptions of the distinctiveness-driven approach.

Within the distinctiveness-driven approach, we found that the similarity and frequency of good stimuli is in fact higher when the substance levels are distributed normally, and the survivability function is concave. This accords well with previous assertions by Unkelbach, Alves, and Koch (2020, for an overview). Moreover, these predictions hold quite well across both absolute and relative baselines.

It is important to emphasize, however, that these insights concern theoretical stringency, not empirical support. We currently lack objective data on the contact base rate or on the typical potency of good and bad stimuli in present or ancestral environments. While experience-sampling approaches to assess differential frequency and similarity provide some insights (e.g., Weitzel & Unkelbach, 2025), they still rely on subjective ratings. The good news is that the relevant parameters may easily be experimentally manipulated using setups like the one introduced in the previous section. Beyond that, adopting the conceptual language proposed here can facilitate clearer comparisons among theoretical models, empirical findings, and research syntheses. This will reduce the need to first determine whether studies are even comparable with one another.

### Limitations and Future Directions

Despite its theoretical and empirical benefits, it is important to acknowledge that our formal account has several limitations on the theoretical level and in its potential to be applied to empirical questions. We introduced multiple simplifying assumptions, which are comprehensively listed in the Appendix. Such simplifications are an inherent part of formalization, as they allow researchers to identify and define the concepts of key importance while maintaining accessibility. Future theoretical or empirical work may relax one or more of these restrictions to adapt the model to specific research questions. When doing this, we recommend making any lifted restrictions explicit to maximize both transparency and comparability with the current work.

Most importantly, we reduced the complexity of our work by focusing exclusively on survival probability as the defining concept of valence, in line with the theories that we started from. In reality, however, people strive for a wide range of potential outcomes such as pleasure, achievement, or social connection (Schwartz & Bilsky, 1987). While it remains unclear how our explanations extend to these other aspects of valence, the current analysis provides a valuable starting point for exploring them.

We also kept several variables in our model constant. For instance, we assumed that all stimuli have the same probability of making contact with a perceiver. This is obviously far from reality: stimuli vary widely in how likely they are to approach a perceiver, probably depending on their level on certain substance dimensions (e.g., their ability to move quickly). However, we aimed to emphasize that not all bad stimuli automatically make contact when not being processed. This insight was not properly accounted for in previous narrative theorizing. To demonstrate this point, it was unnecessary to allow contact probability to vary across stimuli.

In line with the major narrative strands of valence asymmetry explanations, we also neglected any differences between perceivers. Such a differential perspective would need to account for actual differences in the environments in which perceivers have lived in the past or currently live, their current states, and individual biases in their cognitive mechanisms. Relatedly, it would be interesting to consider the possibility that perceivers actively shape or seek out environments (Denrell, 2005; Rauthmann et al., 2015) which we have ignored at the current stage.

In our model, the survival baseline—and, by extension, the valence and potency of encountered stimuli—remains thus fixed and does not adapt to the current state or previous behavior of the perceiver. For example, the value of food remains constant regardless of whether the perceiver is satiated or starving. However, our relative survival baseline, defined as the expected survivability within an environment (i.e., the expected survival probability of a passive perceiver), serves as an approximation to such dynamics: food carries higher value when it is scarce across the environment as a whole. Incorporating additional parameters, such as perceiver-specific survival baselines or situation-dependent contact probabilities, would enhance the model’s flexibility and ecological realism, though at the cost of greatly increased complexity.

Further, the model simplifies the role of learning mechanisms, as these were not the primary focus of the present formalization. This applies to both ontogenetic and phylogenetic learning. Regarding ontogenetic learning, various mechanisms are conceivable: observational learning (e.g., witnessing another person become ill after having contact with a particular fruit), cultural transmission (e.g., being advised by others to avoid a specific type of fruit), or experience-based learning (e.g., becoming ill oneself). Each of these mechanisms may contribute to the ontogenetic adaptation of processing mechanisms in unique ways.

Concerning phylogenetic adaptation, our model simplifies by focusing on survival instead of procreation. There are many instances in which single organisms die to ensure the survival of their species. Think of bees, which die as soon as they have stung a predator. Nevertheless, especially in mammals, survival is an important precondition for procreation. Most importantly, survival is the key variable in Baumeister et al.’s (2001) and Rozin and Royzman’s (2001) approaches. A more nuanced account of gender or age differences in valence asymmetries due to the distinction between procreation and survival, as hinted by Rozin and Royzman (2001), would be an interesting direction for future research.

Finally, we concentrated on a subset of mechanisms within the valence-driven approach. Other concepts, such as contagion, represent promising topics for further formal development.

#### Ecological Validity

A gap remains between the conceptual ideas and how they can be examined in experimental settings. Future studies will need to articulate more clearly how laboratory data are assumed to be connected to real-world outcomes.

Our model offers a framework for operationalizing distinctiveness, valence, and potency in experimental settings. These constructs can be systematically manipulated by altering the substance-level distribution, the survivability function, and the survival baseline. To gain insights into more natural settings, future research may explore methods for assessing these variables in actual environments, beyond the subjective ratings current approaches rely on.

In order to remain faithful to the theoretical reasoning of the approaches analyzed, our model defines valence and potency entirely in terms of survival outcomes. Encountering stimuli and reacting to them in adaptive ways can have dramatic consequences for the perceiver. A starving person may encounter a life-saving nutrient, whereas an overall happy person may suddenly face a deadly threat. Clearly, this poses challenges for empirical translation. No experiment may even remotely match the difference in actual stimulus valence between these situations. For the experimental setting we described, a survival counter combined with performance-contingent financial compensation could serve as a proxy. Alternatively, researchers might use unpleasant but non-harmful stimuli, such as mild electric shocks, or other forms of reward or punishment. Any such choice, however, requires a careful reconsideration of the model and a critical reflection on how closely it maps onto the real-world phenomena in question.

While we subsumed all the different valence asymmetry phenomena—including various negativity and positivity biases—under the general concept of differential processing effort, a comprehensive empirical account should consider the full range of processing domains in which such asymmetries emerge. This could be achieved by varying the experimental paradigms themselves or incorporating additional measures.

#### Positionality, Citations, and Constraints on Generality Statement

We acknowledge that our work is influenced by our personal beliefs and perspectives, as well as the context of the research on which this analysis is based. We collaborated on this project as a group of European researchers of different ages, genders, and experience levels. What unites us is our common belief to improve psychological research by elaborate, precise, and logically stringent theorizing. Most of the empirical work on valence asymmetry has been conducted in Western European or Northern American universities, a privileged population that may be used to encountering an exceptionally high proportion of good stimuli (see also Baumeister et al., 2001). This may have biased both the typical participants’ behavior as well as the researchers’ theorizing and interpretation of empirical results. While we do not claim objectivity in our own perspectives, we believe that by carefully carving out all the underlying theoretical assumptions through formalization, we paved the way to making such potential biases more visible.

## Conclusion

Despite its limitations, we are confident that our formal analysis opens promising avenues for future research on valence asymmetries. By identifying a core set of variables and specifying two distinct explanatory approaches in terms of these variables, our account has deepened the understanding of existing narrative explanations and uncovered previously overlooked assumptions.

Concerning future empirical research, our work offers guidance for operationalizing key constructs and for systematically comparing the two perspectives while ruling out alternative explanations within a coherent empirical framework. A central component of this framework is the deliberate variation of environmental parameters—including those not explicitly addressed but critical to the theories under consideration (e.g., contact probability)—to create a diagnostic testing environment.

In conclusion, our findings highlight that the complexity inherent in such formal accounts is rewarded with the theoretical precision and insight they afford. Future research should build on these insights, extend them to new research questions, and implement them in empirical testing.

## Appendix

### Note on Terminology

We use the term *perceiver* for the typical person who encounters various stimuli and may or may not process their respective properties. We use the term *encounter* to describe the situation in which the perceiver is close enough to a stimulus to detect its properties, but it is not yet clear whether this perceiver will have *contact* C or no contact $\bar{C}$ with this stimulus. Then we distinguish between the probability of surviving the contact with a given stimulus ( P(L|C,S=s) , *survivability*) and the probability that a perceiver will survive the non-contact with any stimulus ( $P(L|\bar{C})$ , *survival baseline*). Survivability is dependent on the *substance level* S=s , that is, the property of a stimulus, and the *survivability function* l(s) . The survival baseline is independent of the substance level of the encountered stimulus. The *valence* V(S=s) of a stimulus follows from the comparison of its survivability and the survival baseline. If the difference between these two is positive, the stimulus is *good*, indicated as G ; if it is negative, the stimulus is *bad*, which is denoted as $\bar{G}$ .

In a given environment, the substance levels of stimuli follow a certain distribution, which we call the *substance distribution* $f_{S}(s)$ . Together with the survival baseline and the survivability function, this results in the *proportion of good stimuli* P(G) , that is, the probability that an encountered stimulus is good. Based on all the parameters introduced and the probability that stimuli make contact by default, which we call *contact probability* P(C) , the probability to survive the typical stimulus encounter in the environment, the *a priori survival probability* $P\left(L|\phi_{0}\right)$ , can be determined.

The probability of surviving with a certain processing mechanism ϕ , the *survival probability with processing effort* P(L|ϕ) , considers the differential *processing effort* E that might be directed towards good P(E|G) and bad $P(E|\bar{G})$ stimuli. *Adaptive advantage* *AA*(φ) is the difference between the survival probability with and without processing effort.

### Notation of Our Parameters

We provide an overview of the notation of all parameters in Table A1 in this Appendix. Note that our notation generally follows probabilistic principles because we consider the main outcomes of our model to be probabilistic. However, to simplify things and reflect the frequency-based reasoning in the distinctiveness-driven approach, we assumed a limited set of stimuli which allowed us to use an arithmetic logic for all operations regarding the distribution, aggregation, and deviation of substance levels. This solely reflects a simplification of the mathematical operations in the paper that is not grounded in theoretical beliefs.

**Table A1.** Meaning and Mathematical Properties of All Model Parameters.

Parameter	Meaning	Narrative term	Mathematical properties			Notes
			Scale	Min	Max	
Stimulus encounters						
L	Survival (outcome)		Dichotomous	0	1	
C	Contact with an encountered stimulus, no contact labeled as $\bar{C}$	Contact	Dichotomous	0	1	
Substance						
S	Substance levels of stimuli	Substance	Interval	0	1	S=s indicates that s a realization of *S*
l(s )	Function yielding P(L|C) from S=s	Survivability function				
$f_{S}(s)$	Frequency distribution of substance levels	Substance distribution				
Valence						
V(S=s)	Valence of a stimulus. V(S=s)>0 labeled as G , “good,” V(S=s)<0 labeled as $\bar{G}$ , “bad”	Valence	Interval	−1	1	
P(C)	Probability that a stimulus gets in contact	Contact probability	Absolute	0	1	
P(G)	Probability that a stimulus is good	Proportion of good stimuli	Absolute	0	1	
Processing						
P(E|G) , $P(E|\bar{G})$	Probability to process a good or bad stimulus	Processing probability	Absolute	0	1	
ϕ	Processing mechanism that produces a certain pattern of P(E|G) and $P(E|\bar{G})$	Processing mechanism	Nominal			
Survival probabilities						
P(L)	Unconditional probability of survival		Absolute	0	1	
P(L|C,S=s)	Probability of surviving the contact with an individual stimulus	Survivability of a stimulus	Absolute	0	1	
$P(L|\bar{C})$	Probability of surviving having no contact with an individual stimulus; independent of its substance	Survival baseline	Absolute	0	1	
P(L|C)	Expected survivability in an environment	Expected survivability	Absolute	0	1	Aggregation across all realizations of *S*
P(L|C,G)	Expected probability of surviving contact with a good stimulus	Expected survivability of good stimuli	Absolute	0	1	Aggregation across all realizations of *S* in the good range(s)
$P(L|C,\bar{G})$	Expected probability of surviving contact with a bad stimulus	Expected survivability of bad stimuli	Absolute	0	1	Aggregation across all realizations of S in the bad range(s)
$P(L|\phi_{0})$	A priori probability to survive a stimulus encounter without processing	A priori survival probability	Absolute	0	1	
P(L|ϕ)	Probability to survive a stimulus encounter with a certain processing mechanism	Survival probability with processing	Absolute	0	1	
Adaptive advantage						
AA(ϕ)	Adaptive advantage obtainable by a certain processing mechanism		Interval	0	1	
Specifications						
Sim(G) , $Sim\left(\bar{G}\right)$	Similarity of good or bad stimuli		Interval	0	1	
dSim	Difference between these similarities	Differential similarity	Interval	−1	1	
dFreq	Differential frequency	Differential frequency	Interval	−1	1	
Pot(G) , $Pot\left(\bar{G}\right)$	Potency of good or bad stimuli		Interval	0	1	
dPot	Difference between these potencies	Differential potency	Interval	−1	1	

*Note.* A comma as in *P*(*L*|*C*, *G*) denotes a joint conditional probability, for example, the probability of survival given the encounter with a good stimulus. An overline as in $\bar{C}$ denotes complement events. For expected survivabilities such as P(L|C) , the conditionality from specific substance levels S=s is removed because when aggregating over randomly distributed variables, the outcome is not conditional on that variable anymore.

### Additional Assumptions and Simplifications

(1) All perceivers are the same. There are no systematic differences in the universe of stimuli that different perceivers are confronted with, or in their processing mechanisms.
(2) Stimuli can only be contacted or not contacted. That is, “dosage” is either 0% or 100%.
(3) The a priori contact probability P(C) is the same for all stimuli. Any possible influence of substance levels on contact probability is ignored.
(4) Substance variables are bounded. Theoretically, they could also be unbounded, but we ignore that possibility here.
(5) Determining the valence of a stimulus requires the same amount of processing for good and bad stimuli. This determination happens first, automatically, and always yields the correct result.
(6) The additional processing that happens afterwards is absolute in the sense that all stimuli of a certain type (good vs. bad) are processed completely.
(7) There are no neutral stimuli. The survivabilities of all stimuli are either above or below the survival baseline (even if only slightly), so that $P\left(\bar{G}\right)=1-P(G)$ .
(8) When additional processing $\left(E\left(G\right),\;E\left(\bar{G}\right)\right)$ happens, it always leads to the optimal result.
(9) The survivability associated with one of the four possible valence and contact combinations ( P(L|C,G ), $P(L|C,\bar{G})$ , $P(L|\bar{C},G$ ), $P(L|\bar{C},\bar{G})$ is the same for all instantiations of the respective combination.
(10) Contiguity is neglected. Consequences always follow immediately from contact or no contact with an encountered stimulus. Of course, processing does not guarantee survival in real life.
(11) Possible gender or age differences regarding which processing mechanisms yield an adaptive advantage are ignored.
(12) As long as a perceiver has contact with a stimulus, no new stimulus is encountered. We ignore the possibility of having contact with several stimuli at the same time.
(13) Valence is one bipolar dimension in the model. We ignore the possibility that there could be two unipolar valence dimensions (one positive and one negative) as suggested by the literature on ambivalence. However, the model allows for multiple bipolar valence dimensions when considering multiple substance dimensions or extending the model to multiple outcomes.
(14) Perceivers stay in their environments. In reality, perceivers move from one environment to another all the time, but we decided to ignore that additional complexity.

## Notes

1. We formalize this by calculating their average deviation and subtracting it from the maximal possible average deviation on the scale with $m(S_{G})$ and $m(S_{\bar{G}})$ denoting the valence-specific means: $Sim\left(G\right)=0.5-\frac{1}{n_{G}}\underset{i\in G}{\sum }|S_{i}-m(S_{G})|$ with $m(S_{G})=\frac{1}{n_{G}}\underset{i\in G}{\sum }S_{i}\;Sim\left(\bar{G}\right)=0.5-\frac{1}{n_{\bar{G}}}\underset{i\in \bar{G}}{\sum }|S_{i}-m(S_{\bar{G}})|$ with $m(S_{\bar{G}})=\frac{1}{n_{\bar{G}}}\underset{i\in \;\bar{G}}{\sum }S_{i}$
